# A catalyst-free multicomponent domino sequence for the diastereoselective synthesis of (*E*)-3-[2-arylcarbonyl-3-(arylamino)allyl]chromen-4-ones

**DOI:** 10.3762/bjoc.10.43

**Published:** 2014-02-21

**Authors:** Pitchaimani Prasanna, Pethaiah Gunasekaran, Subbu Perumal, J Carlos Menéndez

**Affiliations:** 1 Department of Organic Chemistry, School of Chemistry, Madurai Kamaraj University, Madurai - 625 021, Tamilnadu, India; 2 Departamento de Química Orgánica and Farmacéutica, Facultad de Farmacia, Universidad Complutense, 28040 Madrid, Spain

**Keywords:** chromones, domino reactions, Michael additions, multicomponent reactions, transfer hydrogenation

## Abstract

The three-component domino reactions of (*E*)-3-(dimethylamino)-1-arylprop-2-en-1-ones, 3-formylchromone and anilines under catalyst-free conditions afforded a library of novel (*E*)-3-(2-arylcarbonyl-3-(arylamino)allyl)-4*H*-chromen-4-ones in good to excellent yields and in a diastereoselective transformation. This transformation generates one C–C and one C–N bond and presumably proceeds via a reaction sequence comprising a Michael-type addition–elimination reaction, a nucleophilic attack of an enamine to a carbonyl reminiscent of one of the steps of the Bayllis–Hilman condensation, and a final deoxygenation. The deoxygenation is assumed to be induced by carbon monoxide resulting from the thermal decomposition of the dimethylformamide solvent.

## Introduction

Chromones are widely present in nature, especially in the plant kingdom, and a wide variety of useful biological properties are associated with them [1–2]. Chromone derivatives act as effective tyrosine and protein kinase C inhibitors [3] and display antifungal [4–5], antimycobacterial [6], antiviral [7], antihypertensive [8], anti-oxidant [9–12], HIV-inhibitory [13], anti-inflammatory [14–15], immunomodulatory [16], antithrombotic [17], and anticancer [18–20] activities. Furthermore, some chromone derivatives have been identified as suitable fluorophores for live cell imaging [21]. In view of its importance chromone has emerged as a pharmacophore in drug discovery programmes, leading to several drugs in the market (e.g. cromolyn and nedocromil) and thereby continuing to draw the attention of synthetic organic and medicinal chemists [22–24]. However, there are relatively few methods, which allow the preparation of hybrid structures containing chromone derivatives attached to other heterocyclic systems due to the lack of suitable building blocks. In order to come up with such building blocks, we planned the preparation of chromones containing a β-enaminone structural fragment, since enaminones are versatile starting materials in organic synthesis and are notably important for the synthesis of nitrogen heterocycles [25–27]. In particular, β-enaminoketones are endowed with electrophilic and nucleophilic reaction centers and have a versatile reactivity that allows their application in the synthesis of important heterocycles such as indole [28], dihydropyridine [29], quinoline [30], pyrrole [31] and pyridinone [32]. Furthermore, they can take part in one-pot multicomponent reactions with both nucleophilic and electrophilic reactants, leading to a fast access to structurally diverse carbocycles and heterocycles, an area in which we have recently become interested [33–42]. Thus, the present study ia a continuation of our research program on the construction of novel heterocycles employing one-pot green domino-multicomponent transformations [43–54].

**Scheme 1:**
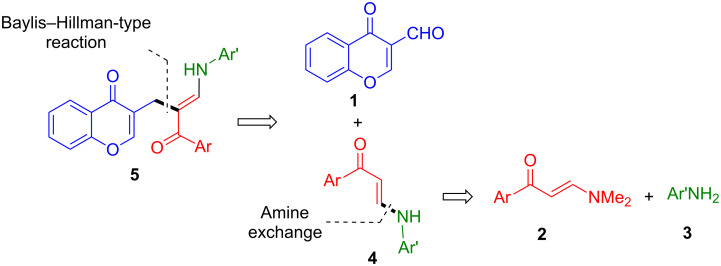
Summary of the transformations involved in the synthesis of compounds **5**, containing chromone and β-enaminoketone moieties.

In order to achieve our goal, we embarked on the study of the three-component reactions between 3-formylchromone (**1**), (*E*)-3-(dimethylamino)-1-arylprop-2-en-1-ones **2** and anilines **3**, which we expected to furnish novel (*E*)-3-[2-arylcarbonyl-3-(arylamino)allyl]-4*H*-chromen-4-ones **5** comprising the desired chromonone and β-enaminoketone moieties via intermediate species **4**. The overall synthetic strategy is summarized in Scheme 1.

## Results and Discussion

We started our study with the optimization of a model reaction between 3-formylchromone (**1**, 1 mmol), (*E*)-3-(dimethylamino)-1-(3-nitrophenyl)prop-2-en-1-one (1 mmol) and 4-methoxyaniline (1 mmol). This three-component reaction was initially examined in solvents such as acetonitrile, dioxane, dimethyl sulfoxide, toluene and ethanol under heating. However, the only isolable product was compound **4d**, which originated from a reaction between the last two components without participation of the chromone substrate (Table 1, entries 1–5). The use of dimethylformamide as a solvent, on the other hand, allowed the isolation of the target (*E*)-3-[3-(4-methoxyphenylamino]-2-(3-nitrobenzoyl)allyl)-4*H*-chromen-4-one (**5f**). After temperature fine-tuning (Table 1, entries 6–11), we identified heating in DMF at 130 °C for 6 h as the optimal conditions, which afforded **5f** in 94% yield (Table 1, entry 11).

**Table 1:** Solvent screen and temperature optimization for the synthesis of compound **5f**.

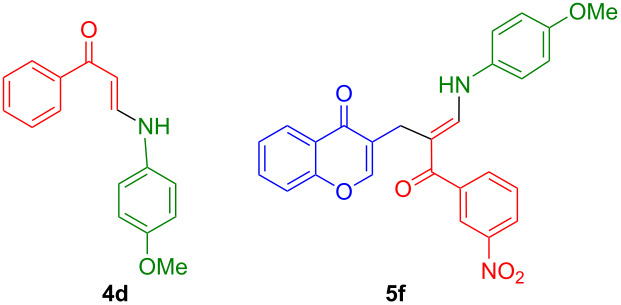				

Entry	Solvent	Time (h)	Temp. (°C)	Yield (%)^a^

1	CH_3_CN	12	80	–^b^
2	dioxane	10	102	–^b^
3	DMSO	10	140	–^b^
4	toluene	10	110	–^b^
5	EtOH	10	78	–^b^
6	DMF	6	60	27
7	DMF	6	80	34
8	DMF	6	100	43
9	DMF	6	110	61
10	DMF	6	120	74
11	DMF	6	130	94

^a^Isolated yield after purification by column chromatography. ^b^Compound **4d** was obtained predominantly.

**Scheme 2:**
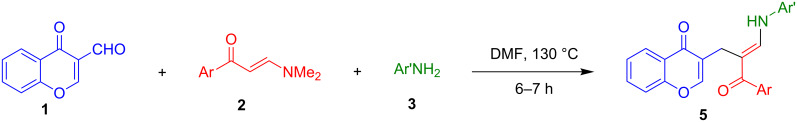
Synthesis of compounds **5**.

All the subsequent reactions of 3-formylchromone (**1**), (*E*)-3-(dimethylamino)-1-arylprop-2-en-1-ones **2** and substituted anilines **3** were performed under the optimal conditions (equimolecular amounts, DMF at 130 °C) and were completed in 6–7 h. After completion of the reaction, removal of the solvent and purification of the residue by column chromatography, (*E*)-3-[2-arylcarbonyl-3-(arylamino)allyl]-4*H*-chromen-4-ones **5** were obtained in pure form in 78–94% yields (Scheme 2 and Table 2). It is noteworthy, that a similar reaction, in which the less hindered, more reactive formaldehyde was employed as the aldehyde component, took a completely divergent course and afforded 5-arylcarbonyl-1,3-diarylhexahydropyrimidines arising from pseudo five-component reactions of (*E*)-3-(dimethylamino)-1-arylprop-2-en-1-ones, two molecules of formaldehyde and two molecules of aniline [55].

**Table 2:** Scope and yields of the synthesis of compounds **5**.

Entry	Comp.	Ar	Ar'	Time (h)	Yield (%)^a^

1	**5a**	C_6_H_5_	4-MeC_6_H_4_	6	85
2	**5b**	4-ClC_6_H_4_	4-MeC_6_H_4_	6	87
3	**5c**	3-NO_2_C_6_H_4_	4-MeC_6_H_4_	6	90
4	**5d**	C_6_H_5_	4-MeOC_6_H_4_	6	89
5	**5e**	4-ClC_6_H_4_	4-MeOC_6_H_4_	6	91
6	**5f**	3-NO_2_C_6_H_4_	4-MeOC_6_H_4_	6	94
7	**5g**	4-ClC_6_H_4_	4-ClC_6_H_4_	6	86
8	**5h**	4-MeC_6_H_4_	4-ClC_6_H_4_	7	84
9	**5i**	4-MeOC_6_H_4_	4-ClC_6_H_4_	7	82
10	**5j**	3-NO_2_C_6_H_4_	4-ClC_6_H_4_	6	87
11	**5k**	C_6_H_5_	4-BrC_6_H_4_	6	80
12	**5l**	4-MeC_6_H_4_	4-BrC_6_H_4_	6	81
13	**5m**	4-MeC_6_H_4_	4-FC_6_H_4_	7	83
14	**5n**	4-MeC_6_H_4_	3-NO_2_C_6_H_4_	7	78
15	**5o**	4-ClC_6_H_4_	C_6_H_5_	6	86
16	**5p**	3-NO_2_C_6_H_4_	C_6_H_5_	6	88

^a^Isolated yield after purification by column chromatography.

**Figure 1:**
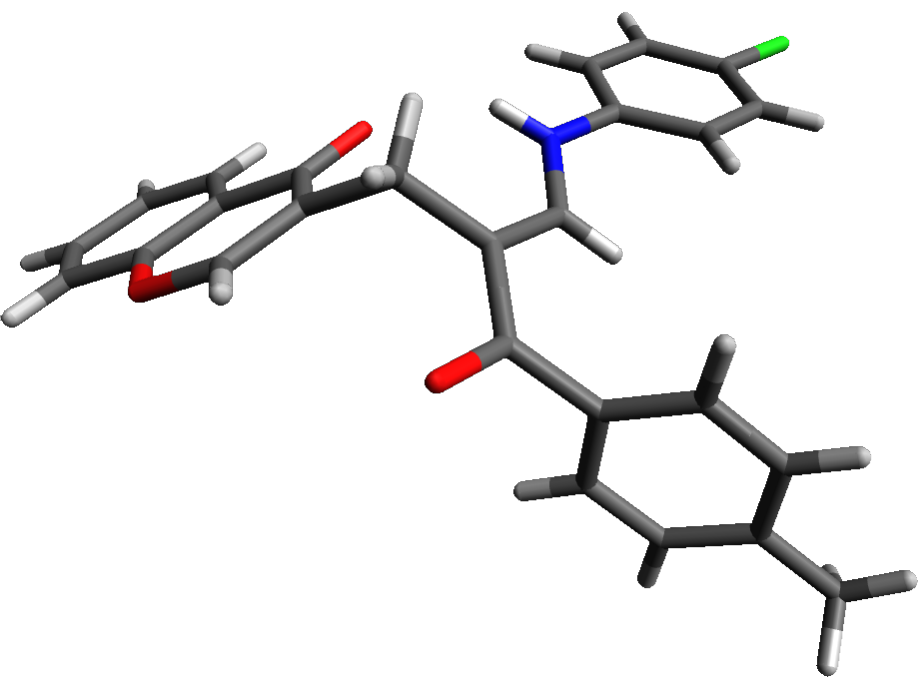
X-ray structure of compound **5h**.

The structure of compounds **5** was deduced from one and two-dimensional NMR spectroscopic data, as detailed in Supporting Information File 1 for **5h** as a representative example. The structure of **5h** deduced from the NMR spectroscopic studies was subsequently confirmed by single-crystal X-ray crystallographic data, as shown in Figure 1 [56].

**Scheme 3:**
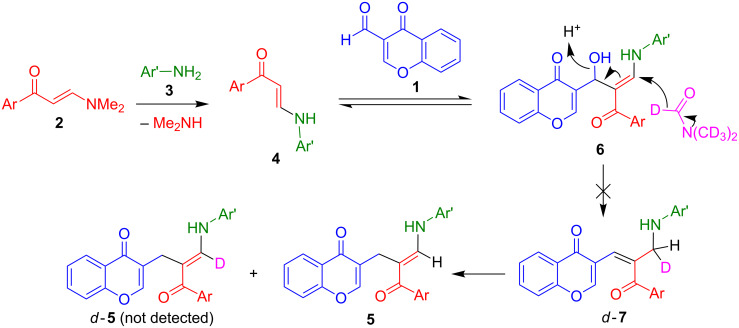
Initial mechanistic proposal to explain the formation of compounds **5** that was ruled out by deuteration experiments.

Our initial mechanistic hypothesis accounting for the formation of compounds **5** is depicted in Scheme 3. An initial Michael addition–elimination reaction, leading to the exchange between the starting arylamine **3** and dimethylamine, explains the formation of intermediates **4**, which were the final reaction products in most investigated solvents. However, in DMF, the enamine moiety of **4** attacks the aldehyde group in **1** giving rise to **6**. This reaction resembles one of the steps of the Bayllis–Hilman condensation and is presumably promoted by the presence of traces of formic acid as a contaminant of the solvent [57].

We established the feasibility of the first step by showing that the reaction between two of our starting enaminones, namely **2a** (Ar = Ph) and **2c** (Ar = 3-NO_2_C_6_H_4_) with aniline under our standard reaction conditions (DMF, 130 °C) affords the corresponding compounds **4** after 3 h in 94% and 90% yields, respectively. As previously mentioned (Table 1), the nature of the solvent was found to be of critical importance, and the transformation of intermediate **4** into the final products **5** was found to work only in DMF, among many investigated solvents. Since the reaction between **4** and **1** is proposed to be catalyzed by acid, we carried out the multicomponent reaction in a selection of solvents (dioxane, acetonitrile, dimethyl sulfoxide, ethanol, ethylene glycol) in the presence of one equivalent of HCl, but all these attempts failed, while the same conditions were successful in DMF. These results can be explained by assuming that the initial aldol-type reaction between **1** and **4** to give **6** is reversible and is driven to completion by the reduction step, which takes place in DMF only. Thus, interestingly, the reaction does not stop at compound **6**, but instead undergoes a reductive termination step, which leads to the final products **5**. This reduction step is intriguing, and a first mechanistic possibility could be a hydride transfer from formate anion. While this mechanism might partly account for the observed reduction, we acknowledge that it is problematic to rely on solvent impurities to account for a stoichiometric reduction. Alternatively, dimethylformamide itself might have been the reducing agent. There are a few scattered literature reports on the use of DMF as a reducing agent, the first of which seems to be the reduction of diazonium tetrafluoroborate salts to arenes [58]. DMF has also been described as a reducing agent acting by hydride transfer in Pd-catalyzed processes, where the metal plays a critical role in the reduction by decomposing it and facilitating hydride transfer from a Pd intermediate [59–61]. However, it is not clear whether the same type of reaction may take place under our conditions. In order to test this ideas experimentally, we carried out the reaction between 3-formylchromone (**1**), (*E*)-3-(dimethylamino)-1-(4-chlorophenyl)prop-2-en-1-one and *p*-toluidine in DMF-*d*_7_. If the mechanistic hypothesis was correct, this reaction should lead to a *d*-**7** intermediate and hence to a mixture of **5** and *d*-**5**, with the latter being major due to isotope effects in the tautomeric equilibrium. However, this reaction failed to show any incorporation of deuterium into **5**, and therefore we had to abandon the hydride-transfer hypothesis.

**Scheme 4:**
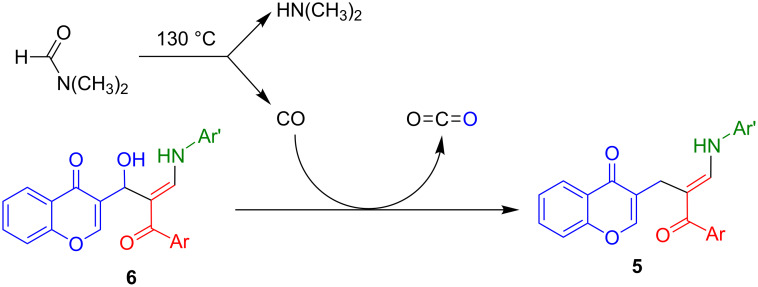
Alternative mechanistic proposal based on a carbon monoxide-induced deoxygenation.

We came up with an alternative explanation based on the well-known fact that DMF decomposes into dimethylamine and carbon monoxide at its boiling point. Carbon monoxide can act as a deoxygenating agent [62] and thus explain the transformation of **6** into **5** as shown in Scheme 4. Because of the synthetic interest of allylic and benzylic deoxygenations, further research into this reaction is under way in our laboratories.

## Conclusion

We have developed a facile three-component diastereoselective synthesis of novel (*E*)-3-[2-arylcarbonyl-3-(arylamino)allyl]-4*H*-chromen-4-ones containing chromone and β-enaminoketone structural fragments from simple, readily available starting materials in a one-pot operation and in good to excellent yields. This transformation occurs via a domino sequence of reactions, which generates one C–C and one C–N bond. Presumably, this transformation proceeds via a reaction sequence comprising a Michael-type addition–elimination reaction, a nucleophilic attack of an enamine to a carbonyl, and a final deoxygenation step. We propose that the deoxygenation step is induced by carbon monoxide resulting from thermal decomposition of the dimethylformamide solvent.

## Experimental

**General methods.** Melting points were measured in open capillary tubes and are uncorrected. The ^1^H NMR, ^13^C NMR, DEPT, H,H-COSY, C,H-COSY and HMBC spectra were recorded on a Bruker (Avance) 300 MHz NMR instrument by using TMS as an internal standard and CDCl_3_ as a solvent. Standard Bruker software was used throughout. Chemical shifts are given in parts per million (δ-scale), and the coupling constants are given in Hertz. Silica gel-G plates (Merck) were used for TLC analysis with a mixture of petroleum ether (60–80 °C) and ethyl acetate as an eluent. Elemental analyses were performed on a Perkin Elmer 2400 Series II Elemental CHNS analyzer.

**General procedure for the synthesis of (*E*)-3-(2-arylcarbonyl-3-(arylamino)allyl)-4*H*-chromen-4-one derivatives 5a–5p**. In a similar manner as described in [55], a mixture of 3-formylchromone (**1**, 1 mmol), enaminone **2** (1 mmol) and aniline **3** (1 mmol) in DMF (5 mL) was heated at 130 °C for 6–7 h. The reaction progress was monitored by TLC. After completion of the reaction, the solvent was removed and the product was purified by column chromatography with a petroleum ether–ethyl acetate mixture (4:1 v/v) as an eluent to afford compounds **5**. Characterization data for representative compounds are given below. The characterization data for the full library can be found in Supporting Information File 1.

***(E)-3-(2-Benzoyl-3-(p-tolylamino)allyl)-4H-chromen-4-one* (5a):** Isolated yield 0.336 g (85%); colorless solid; mp 215–216 °C; ^1^H NMR (300 MHz, CDCl_3_, δ, ppm) 2.28 (s, 3H), 3.71 (s, 2H), 6.92 (d, *J* = 8.4 Hz, 2H), 7.08 (d, *J* = 8.1 Hz, 2H), 7.37–7.45 (m, 4H), 7.47–7.56 (m, 4H), 7.66–7.71 (m, 1H), 8.31 (d, *J* = 7.8 Hz, 1H), 8.52 (s, 1H), 9.93 (d, *J* = 12.6 Hz, 1H); ^13^C NMR (75 MHz, CDCl_3_, δ, ppm): 20.1, 20.6, 113.3, 115.8, 118.3, 123.7, 123.9, 125.0, 125.9, 128.0, 128.5, 129.8, 130.1, 132.3, 133.7, 138.9, 140.9, 146.6, 156.2, 156.7, 179.8, 195.0; HRMS–ESI (*m*/*z*): [M − H]^−^ calcd for C_26_H_20_NO_3_, 394.14432; found, 394.144817; Anal. calcd for C_26_H_21_NO_3_: C, 78.97; H, 5.35; N, 3.54; found: C, 79.02; H, 5.29; N, 3.62.

***(E)-3-(2-(3-Nitrobenzoyl)-3-(p-tolylamino)allyl)-4H-chromen-4-one* (5c):** Isolated yield 0.396 g (90%); yellow solid; mp 206–207 °C; ^1^H NMR (300 MHz, CDCl_3_, δ, ppm) 2.32 (s, 3H), 3.72 (s, 2H), 6.93 (d, *J* = 8.4 Hz, 2H), 7.10 (d, *J* = 8.1 Hz, 2H), 7.40–753 (m, 3H), 7.59 (d, *J* = 8.0 Hz, 1H), 7.68–7.74 (m, 1H), 7.81 (d, *J* = 7.5 Hz, 1H), 8.28–8.35 (m, 3H), 8.51 (s, 1H), 10.2 (d, *J* = 12.6 Hz, 1H); ^13^C NMR (75 MHz, CDCl_3_, δ, ppm) 20.1, 20.7, 113.0, 116.2, 118.4, 123.3, 123.6, 123.7, 124.4, 125.2, 125.9, 129.2, 130.2, 133.1, 133.9, 134.1, 138.4, 142.5, 147.3, 148.0, 156.1, 156.7, 179.9, 191.8; HRMS–ESI (*m*/*z*): [M − H]^−^ calcd for C_26_H_19_N_2_O_5_, 439.12940; found, 439.12995; Anal. calcd for C_26_H_20_N_2_O_5_: C, 70.90; H, 4.58; N, 6.36; found: C, 70.84; H, 4.62; N, 6.29.

***(E)-3-(3-(4-Chlorophenylamino)-2-(4-methylbenzoyl)allyl)-4H-chromen-4-one* (5h):** Isolated yield 0.361 g (84%); colorless solid; mp 219–220 °C; ^1^H NMR (300 MHz, CDCl_3_, δ, ppm) 2.40 (s, 3H), 3.70 (s, 2H), 6.95 (d, *J* = 8.7 Hz, 2H), 7.19–7.26 (m, 4H), 7.40–7.46 (m, 3H), 7.48–7.52 (m, 2H), 7.69 (t, *J* = 7.1 Hz, 1H), 8.30 (d, *J* = 7.2 Hz, 1H), 8.51 (s, 1H), 10.04 (d, *J* = 12.3 Hz, 1H); ^13^C NMR (75 MHz, CDCl_3_, δ, ppm) 20.4, 21.4, 114.4, 116.8, 118.4, 123.7, 125.1, 125.8, 127.4, 128.6, 128.8, 129.5, 133.8, 137.7, 140.1, 140.4, 145.3, 156.3, 156.7, 179.9, 195.2; HRMS–ESI (*m*/*z*): [M − H]^−^ calcd for C_26_H_19_ClNO_3_, 428.10535; found, 428.10589; Anal. calcd for C_26_H_20_ClNO_3_: C, 72.64; H, 4.69; N, 3.26; found: C, 72.57; H, 4.75; N, 3.31.

***(E)-3-(3-(4-Bromophenylamino)-2-(4-methylbenzoyl)allyl)-4H-chromen-4-one* (5l):** Isolated yield 0.384 g (81%); colorless solid; mp 223–224 °C; ^1^H NMR (300 MHz, CDCl_3_, δ, ppm) 2.40 (s, 3H), 3.70 (s, 2H), 6.90 (d, *J* = 8.7 Hz, 2H), 7.21 (d, *J* = 8.1 Hz, 2H), 7.36–7.41 (m, 3H), 7.43–7.52 (m, 4H), 7.69 (dd, *J* = 7.5 Hz, 1.5 Hz, 1H), 8.30 (dd, *J* = 8.1 Hz, 1.8 Hz, 1H), 8.51 (s, 1H), 10.04 (d, *J* = 12.0 Hz, 1H); ^13^C NMR (75 MHz, CDCl_3_, δ, ppm) 20.4, 21.4, 114.5, 114.7, 117.2, 118.4, 123.6, 125.1, 125.8, 128.6, 128.8, 132.5, 133.8, 137.7, 140.4, 140.6, 145.1, 156.4, 156.7, 179.9, 195.2; HRMS–ESI (*m*/*z*): [M − H]^−^ calcd for C_26_H_19_BrNO_3_, 472.05483; found, 472.05538; Anal. calcd for C_26_H_20_BrNO_3_: C, 65.83; H, 4.25; N, 2.95; found: C, 65.70; H, 4.34; N, 2.91.

***(E)-3-(2-(4-Chlorobenzoyl)-3-(phenylamino)allyl)-4H-chromen-4-one* (5o):** Isolated yield 0.357 g (86%); colorless solid; mp 245–246 °C; ^1^H NMR (300 MHz, CDCl_3_, δ, ppm) 3.71 (s, 2H), 7.00–7.05 (m, 3H), 7.26–7.33 (m, 2H), 7.37–7.40 (m, 2H), 7.44–7.55 (m, 5H), 7.67–7.72 (m, 1H), 8.31 (d, *J* = 7.8 Hz, 1H), 8.50 (s, 1H), 10.08 (d, *J* = 12.3 Hz, 1H); ^13^C NMR (75 MHz, CDCl_3_, δ, ppm) 20.2, 113.7, 115.9, 118.4, 122.9, 123.7, 125.1, 125.9, 128.4, 129.7, 129.9, 133.8, 136.1, 139.2, 141.1, 146.2, 156.2, 156.7, 179.8, 193.8; HRMS–ESI (*m*/*z*): [M − H]^−^ calcd for C_25_H_17_ClNO_3_, 414.08970; found, 414.09024; Anal. calcd for C_25_H_18_ClNO_3_: C, 72.20; H, 4.36; N, 3.37; found: C, 72.09; H, 4.24; N, 3.41.

**General procedure for the isolation of intermediates 4**. A mixture of the suitable enaminone **2** (1 mmol) and aniline **3** (1 mmol) in DMF (5 mL) was heated at 130 °C for 3 h. The reaction progress was monitored by TLC. After completion of the reaction, the solvent was removed and the product was purified by column chromatography with a petroleum ether–ethyl acetate mixture (4:1 v/v) as an eluent. Characterization data for compounds **4** can be found in Supporting Information File 1.

## Supporting Information

File 1 Experimental details, full characterization data, detailed structural characterization of compound **5h** and copies of the spectra of all compounds.
