# Supplementary material for: A catalyst-free multicomponent domino sequence for the diastereoselective synthesis of (E)-3-[2-arylcarbonyl-3-(arylamino)allyl]chromen-4-ones
Source: Beilstein J Org Chem. 2014 Feb 21;10:459–65. doi: 10.3762/bjoc.10.43 (PMC3944430; doi:10.3762/bjoc.10.43)
Supplement: File 1 — Experimental details, full characterization data, detailed structural characterization of compound 5h and copies of the spectra of all compounds. [file Beilstein_J_Org_Chem-10-459-s001.pdf]

Supporting Information

for

A catalyst-free multicomponent domino sequence for the diastereoselective synthesis of (*E*)-3-[2-arylcarbonyl-3-(arylamino)allyl]chromen-4-ones

Pitchaimani Prasanna[1], Pethaiah Gunasekaran[1], Subbu Perumal*[1] and J. Carlos Menéndez*[2]

Address: [1]Department of Organic Chemistry, School of Chemistry, Madurai Kamaraj University, Madurai - 625 021, Tamilnadu, India and [2]Departamento de Química Orgánica and Farmacéutica, Facultad de Farmacia, Universidad Complutense, 28040 Madrid, Spain

Email: Subbu Perumal - subbu.perum@gmail.com; J. Carlos Menéndez - josecm@farm.ucm.es

*Corresponding author

Experimental details, full characterization data, detailed structural characterization of compound **5h** and copies of the spectra of all compounds

1. Experimental details and full characterization data

General experimental methods. Melting points were measured in open capillary tubes and are uncorrected. The ^{1}H NMR, ^{13}C NMR, DEPT, H,H-COSY, C,H-COSY and HMBC spectra were recorded on a Bruker (Avance) 300 MHz NMR instrument using TMS as internal standard and $CDCl_3$ as solvent. Standard Bruker software was used throughout. Chemical shifts are given in parts per million (δ-scale) and the coupling constants are given in Hertz. Silica gel-G plates (Merck) were used for TLC analysis with a mixture of petroleum ether (60–80 °C) and ethyl acetate as eluent. Elemental analyses were performed on a Perkin Elmer 2400 Series II Elemental CHNS analyzer.

General procedure for the synthesis of (*E*)-3-(2-arylcarbonyl-3-(arylamino)allyl)-4*H*-chromen-4-one 5a–5p. A mixture of 3-formylchromone (**1**, 1 mmol), enaminone **2** (1 mmol) and aniline **3** (1 mmol) in DMF (5 mL) was heated at 130 °C for 6–7 h. The reaction progress was monitored by thin-layer chromatography. After completion of the reaction, the solvent was removed and the product was purified by column chromatography using petroleum ether–ethyl acetate mixture (4:1 v/v) as eluent to afford (*E*)-3-(2-arylcarbonyl-3-(arylamino)allyl)-4*H*-chromen-4-ones **5**.

***(E)-3-(2-Benzoyl-3-(p-tolylamino)allyl)-4H-chromen-4-one* (*5a*):** Isolated yield 0.336 g (85%); Colorless solid; m.p. 215-216 ^{o}C. ^{1}H NMR (300 MHz, $CDCl_3$, δ ppm): 2.28 (s, 3H), 3.71 (s, 2H), 6.92 (d, *J* = 8.4 Hz, 2H), 7.08 (d, *J* = 8.1 Hz, 2H), 7.37-7.45 (m, 4H), 7.47-7.56 (m, 4H), 7.66-7.71 (m, 1H), 8.31 (d, *J* = 7.8 Hz, 1H), 8.52 (s, 1H), 9.93 (d, *J* = 12.6 Hz, 1H); ^{13}C NMR (75 MHz, $CDCl_3$, δ ppm): 20.1, 20.6, 113.3, 115.8, 118.3, 123.7, 123.9, 125.0, 125.9, 128.0, 128.5, 129.8, 130.1, 132.3, 133.7, 138.9, 140.9, 146.6, 156.2, 156.7, 179.8, 195.0; HRMS (ESI) calcd for $C_{26}H_{20}NO_3$ [M - H]$^-$ 394.14432; found 394.144817. Anal. Calcd for $C_{26}H_{21}NO_3$: C, 78.97; H, 5.35; N, 3.54. Found: C, 79.02; H, 5.29; N, 3.62.

***(E)-3-(2-(4-Chlorobenzoyl)-3-(p-tolylamino)allyl)-4H-chromen-4-one* (*5b*):** Isolated yield 0.374 g (87%); Colorless solid; m.p. 250-251 ^{o}C. ^{1}H NMR (300 MHz, $CDCl_3$, δ ppm): 2.30 (s, 3H), 3.69 (s, 2H), 6.93 (d, *J* = 8.4 Hz, 2H), 7.10 (d, *J* = 8.1 Hz, 2H), 7.35-7.40 (m, 2H), 7.43-7.48 (m, 4H), 7.51 (s, 1H), 7.69 (td, *J* = 9 Hz, 1.8 Hz, 1H), 8.30 (dd, *J* = 6.0 Hz, 1.5 Hz, 1H), 8.50 (s, 1H), 10.0 (d, *J* = 12.6 Hz, 1H); ^{13}C NMR (75 MHz, $CDCl_3$, δ ppm): 20.2, 20.7, 113.2, 115.9, 118.3, 123.6, 123.7, 125.1, 125.9, 128.3, 129.9, 130.1, 132.6, 133.8, 136.0, 138.7, 139.3, 146.6, 156.1, 156.7, 179.8, 193.6; HRMS (ESI) calcd for $C_{26}H_{19}ClNO_3$ $[M - H]^-$ 428.10535; found 428.10589. Anal. Calcd for $C_{26}H_{20}ClNO_3$: C, 72.64; H, 4.69; N, 3.26. Found: C, 72.77; H, 4.77; N, 3.15.

***(E)-3-(2-(3-Nitrobenzoyl)-3-(p-tolylamino)allyl)-4H-chromen-4-one* (*5c*):** Isolated yield 0.396 g (90%); Yellow solid; m.p. 206-207 ^{o}C. ^{1}H NMR (300 MHz, $CDCl_3$, δ ppm): 2.32 (s, 3H), 3.72 (s, 2H), 6.93 (d, *J* = 8.4 Hz, 2H), 7.10 (d, *J* = 8.1 Hz, 2H), 7.40-753 (m, 3H), 7.59 (d, *J* = 8.0 Hz, 1H), 7.68-7.74 (m, 1H), 7.81 (d, *J* = 7.5 Hz, 1H), 8.28-8.35 (m, 3H), 8.51 (s, 1H), 10.2 (d, *J* = 12.6 Hz, 1H); ^{13}C NMR (75 MHz, $CDCl_3$, δ ppm): 20.1, 20.7, 113.0, 116.2, 118.4, 123.3, 123.6, 123.7, 124.4, 125.2, 125.9, 129.2, 130.2, 133.1, 133.9, 134.1, 138.4, 142.5, 147.3, 148.0, 156.1, 156.7, 179.9, 191.8; HRMS (ESI) calcd for $C_{26}H_{19}N_2O_5$ $[M - H]^-$ 439.12940; found 439.12995. Anal. Calcd for $C_{26}H_{20}N_2O_5$: C, 70.90; H, 4.58; N, 6.36. Found: C, 70.84; H, 4.62; N, 6.29.

***(E)-3-(2-Benzoyl-3-(4-methoxyphenylamino)allyl)-4H-chromen-4-one* (*5d*):** Isolated yield 0.392 g (89%); Colorless solid; m.p. 212-213 ^{o}C. ^{1}H NMR (300 MHz, $CDCl_3$, δ ppm): 3.71 (s, 2H), 3.77 (s, 3H), 6.83 (d, *J* = 9.0 Hz, 2H), 6.96 (d, *J* = 8.7 Hz, 2H), 7.35-7.43 (m, 4H), 7.45-7.51 (m, 4H), 7.66-7.71 (m, 1H), 8.29-8.32 (m, 1H), 8.53 (s, 1H), 9.90 (d, *J* = 12.6 Hz, 1H); ^{13}C NMR (75 MHz, $CDCl_3$, δ ppm): 20.1, 55.6, 112.9, 115.0, 117.2, 118.3, 123.7, 123.9, 125.0, 125.8, 128.0, 128.4, 129.7, 133.7, 135.0, 141.0, 147.0, 155.7, 156.2, 156.7, 179.8, 194.8; HRMS (ESI) calcd for $C_{26}H_{20}NO_4$ $[M - H]^-$ 410.13923; found

410.13978. Anal. Calcd for $C_{26}H_{21}NO_4$: C, 75.90; H, 5.14; N, 3.40. Found: C, 75.79; H, 5.05; N, 3.46.

***(E)-3-(2-(4-Chlorobenzoyl)-3-(4-methoxyphenylamino)allyl)-4H-chromen-4-one* (*5e*):** Isolated yield 0.405 g (91%); Colorless solid; m.p. 237-238 ^{o}C. ^{1}H NMR (300 MHz, $CDCl_3$, δ ppm): 3.69 (s, 2H), 3.78 (s, 3H), 6.84-6.88 (m, 2H), 6.95-6.99 (m, 2H), 7.35-7.38 (m, 2H), 7.41-7.46 (m, 4H), 7.50 (d, *J* = 8.4 Hz, 1H), 7.67-7.72 (td, *J* = 7.5 Hz, 1.7 Hz, 1H), 8.30 (dd, *J* = 6.0 Hz, 1.5 Hz, 1H), 8.50 (s, 1H), 9.97 (d, *J* = 12.3 Hz, 1H); ^{13}C NMR (75 MHz, $CDCl_3$, δ ppm): 20.2, 55.6, 112.8, 115.1, 117.3, 118.4, 123.7, 123.8, 125.1, 125.8, 128.3, 129.8, 133.8, 134.8, 135.9, 139.4, 147.1, 155.9, 156.2, 156.7, 179.9, 193.4; HRMS (ESI) calcd for $C_{26}H_{19}ClNO_4$ $[M - H]^-$ 444.10026; found 444.10081. Anal. Calcd for $C_{26}H_{20}ClNO_4$: C, 70.03; H, 4.52; N, 3.14. Found: C, 69.91; H, 4.56; N, 3.23.

***(E)-3-(3-(4-Methoxyphenylamino)-2-(3-nitrobenzoyl)allyl)-4H-chromen-4-one* (*5f*):** Isolated yield 0.429 g (94%); Orange solid; m.p. 209-210 ^{o}C. ^{1}H NMR (300 MHz, $CDCl_3$, δ ppm): 3.72 (s, 2H), 3.77 (s, 3H), 6.85 (d, *J* = 8.7 Hz, 2H), 6.98 (d, *J* = 8.7 Hz, 2H), 7.35-7.47 (m, 2H), 7.52 (d, *J* = 8.4 Hz, 1H), 7.59 (t, *J* = 8.0 Hz, 1H), 7.71 (t, *J* = 7.7 Hz, 1H), 7.82 (d, *J* = 7.5 Hz, 1H), 8.28-8.35 (m, 3H), 8.51 (s, 1H), 10.18 (d, *J* = 12.6 Hz, 1H); ^{13}C NMR (75 MHz, $CDCl_3$, δ ppm): 20.1, 55.6, 112.6, 115.1, 117.6, 118.4, 123.2, 123.5, 123.6, 124.3, 125.2, 125.8, 129.2, 133.9, 134.1, 134.5, 142.6, 147.7, 148.0, 156.1, 156.2, 156.7, 179.9, 191.6; HRMS (ESI) calcd for $C_{26}H_{19}N_2O_6$ $[M - H]^-$ 455.12431; found 455.12486. Anal. Calcd for $C_{26}H_{20}N_2O_6$: C, 68.42; H, 4.42; N, 6.14. Found: C, 68.50; H, 4.38; N, 6.04.

***(E)-3-(2-(4-Chlorobenzoyl)-3-(4-chlorophenylamino)allyl)-4H-chromen-4-one* (*5g*):** Isolated yield 0.387 g (86%); Colorless solid; m.p. 260-261 ^{o}C. ^{1}H NMR (300 MHz, $CDCl_3$, δ ppm): 3.69 (s, 2H), 6.94-6.97 (m, 2H), 7.23-7.27 (m, 2H), 7.37-7.42 (m, 3H), 7.43-7.47 (m, 3H), 7.51 (d, *J* = 8.4 Hz, 1H), 7.70 (td, *J* = 7.5 Hz, 1.8 Hz, 1H), 8.30 (dd, *J* = 9 Hz, 1.5 Hz, 1H), 8.49 (s, 1H), 10.15 (d, *J* = 12.3 Hz, 1H); ^{13}C NMR (75 MHz, $CDCl_3$, δ ppm): 20.3,

114.2, 117.0, 118.4, 123.5, 123.6, 125.2, 125.8, 127.8, 128.4, 129.7, 129.9, 133.9, 136.3, 139.0, 139.9, 145.7, 156.3, 156.7, 179.9, 193.8; HRMS (ESI) calcd for $C_{25}H_{16}Cl_2NO_3$ [M - H]$^-$ 448.05072; found 448.05127. Anal. Calcd for $C_{25}H_{17}Cl_2NO_3$: C, 66.68; H, 3.81; N, 3.11. Found: C, 66.81; H, 3.74; N, 3.22.

***(E)-3-(3-(4-Chlorophenylamino)-2-(4-methylbenzoyl)allyl)-4H-chromen-4-one* (5*h*):** Isolated yield 0.361 g (84%); Colorless solid; m.p. 219-220 ^oC. ^1H NMR (300 MHz, $CDCl_3$, δ ppm): 2.40 (s, 3H), 3.70 (s, 2H), 6.95 (d, *J* = 8.7 Hz, 2H), 7.19-7.26 (m, 4H), 7.40-7.46 (m, 3H), 7.48-7.52 (m, 2H), 7.69 (t, *J* = 7.1 Hz, 1H), 8.30 (d, *J* = 7.2 Hz, 1H), 8.51 (s, 1H), 10.04 (d, *J* = 12.3 Hz, 1H); ^{13}C NMR (75 MHz, $CDCl_3$, δ ppm): 20.4, 21.4, 114.4, 116.8, 118.4, 123.7, 125.1, 125.8, 127.4, 128.6, 128.8, 129.5, 133.8, 137.7, 140.1, 140.4, 145.3, 156.3, 156.7, 179.9, 195.2; HRMS (ESI) calcd for $C_{26}H_{19}ClNO_3$ [M - H]$^-$ 428.10535; found 428.10589. Anal. Calcd for $C_{26}H_{20}ClNO_3$: C, 72.64; H, 4.69; N, 3.26. Found: C, 72.57; H, 4.75; N, 3.31.

***(E)-3-(3-(4-Chlorophenylamino)-2-(4-methoxybenzoyl)allyl)-4H-chromen-4-one* (5*i*):** Isolated yield 0.365 g (82%); Colorless solid; m.p. 195-196 ^oC. ^1H NMR (300 MHz, $CDCl_3$, δ ppm): 3.70 (s, 2H), 3.86 (s, 3H), 6.91 (d, *J* = 8.7 Hz, 2H), 6.96 (d, *J* = 8.7 Hz, 2H), 7.23-7.26 (m, 2H), 7.41-7.52 (m, 5H), 7.67-7.72 (m, 1H), 8.30 (dd, *J* = 6.0 Hz, 1.2 Hz, 1H), 8.51 (s, 1H), 10.01 (d, *J* = 12.0 Hz, 1H); ^{13}C NMR (75 MHz, $CDCl_3$, δ ppm): 20.6, 55.4, 113.5, 114.3, 116.8, 118.4, 123.7, 125.1, 125.8, 127.3, 129.6, 130.5, 133.8, 140.2, 144.8, 156.3, 156.7, 161.5, 179.9, 194.5; HRMS (ESI) calcd for $C_{26}H_{19}ClNO_4$ [M - H]$^-$ 444.10026; found 444.10081. Anal. Calcd for $C_{26}H_{20}ClNO_4$: C, 70.03; H, 4.52; N, 3.14. Found: C, 69.98; H, 4.59; N, 3.05.

***(E)-3-(3-(4-Chlorophenylamino)-2-(3-nitrobenzoyl)allyl)-4H-chromen-4-one* (5*j*):** Isolated yield 0.401 g (87%); Yellow solid; m.p. 227-228 ^oC. ^1H NMR (300 MHz, $CDCl_3$, δ ppm): 3.72 (s, 2H), 6.96 (d, *J* = 8.7 Hz, 2H), 7.25 (d, *J* = 8.7 Hz, 2H), 7.35-7.54 (m, 3H),

7.61 (t, *J* = 7.8 Hz, 1H), 7.70-7.75 (m, 1H), 7.82 (d, *J* = 7.5 Hz, 1H), 8.30-8.35 (m, 3H), 8.51 (s, 1H), 10.37 (d, *J* = 12.3 Hz, 1H); ^{13}C NMR (75 MHz, $CDCl_3$, δ ppm): 20.3, 114.1, 117.2, 118.4, 123.2, 123.3, 123.6, 124.6, 125.3, 125.8, 128.3, 129.3, 129.7, 134.0, 139.6, 142.2, 146.4, 148.1, 156.3, 156.7, 180.0, 192.1; HRMS: calcd. for $C_{25}H_{16}ClN_2O_5$ [M - H]$^-$ 459.07477; found 459.07532. Anal. Calcd for $C_{25}H_{17}ClN_2O_5$: C, 65.15; H, 3.72; N, 6.08. Found: C, 65.24; H, 3.68; N, 5.97.

***(E)-3-(2-Benzoyl-3-(4-bromophenylamino)allyl)-4H-chromen-4-one* (*5k*):** Isolated yield 0.368 g (80%); Colorless solid; m.p. 231-232 ^{o}C. ^{1}H NMR (300 MHz, $CDCl_3$, δ ppm): 3.70 (s, 2H), 6.89 (d, *J* = 8.7 Hz, 2H), 7.36-7.40 (m, 3H), 7.43-7.46 (m, 3H), 7.48-7.52 (m, 4H), 7.67-7.73 (m, 1H), 8.30 (dd, *J* = 6.0 Hz, 1.5 Hz, 1H), 8.52 (s, 1H), 10.09 (d, *J* = 12.3 Hz, 1H); ^{13}C NMR (75 MHz, $CDCl_3$, δ ppm): 20.3, 114.5, 114.9, 117.3, 118.4, 123.6, 125.2, 125.8, 128.1, 128.4, 130.1, 132.5, 133.9, 140.5, 140.6, 145.5, 156.3, 156.7, 179.9, 195.2; HRMS (ESI) calcd for $C_{25}H_{17}BrNO_3$ [M - H]$^-$ 458.03918; found 458.03973. Anal. Calcd for $C_{25}H_{18}BrNO_3$: C, 65.23; H, 3.94; N, 3.04. Found: C, 65.35; H, 4.01; N, 2.98.

***(E)-3-(3-(4-Bromophenylamino)-2-(4-methylbenzoyl)allyl)-4H-chromen-4-one* (*5l*):** Isolated yield 0.384 g (81%); Colorless solid; m.p. 223-224 ^{o}C. ^{1}H NMR (300 MHz, $CDCl_3$, δ ppm): 2.40 (s, 3H), 3.70 (s, 2H), 6.90 (d, *J* = 8.7 Hz, 2H), 7.21 (d, *J* = 8.1 Hz, 2H), 7.36-7.41 (m, 3H), 7.43-7.52 (m, 4H), 7.69 (dd, *J* = 7.5 Hz, 1.5 Hz, 1H), 8.30 (dd, *J* = 8.1 Hz, 1.8 Hz, 1H), 8.51 (s, 1H), 10.04 (d, *J* = 12.0 Hz, 1H); ^{13}C NMR (75 MHz, $CDCl_3$, δ ppm): 20.4, 21.4, 114.5, 114.7, 117.2, 118.4, 123.6, 125.1, 125.8, 128.6, 128.8, 132.5, 133.8, 137.7, 140.4, 140.6, 145.1, 156.4, 156.7, 179.9, 195.2; HRMS (ESI) calcd for $C_{26}H_{19}BrNO_3$ [M - H]$^-$ 472.05483; found 472.05538. Anal. Calcd for $C_{26}H_{20}BrNO_3$: C, 65.83; H, 4.25; N, 2.95. Found: C, 65.70; H, 4.34; N, 2.91.

(E)*-3-(3-(4-Fluorophenylamino)-2-(4-methylbenzoyl)allyl)-4*H*-chromen-4-one** (**5*m): Isolated yield 0.343 g (83%); Colorless solid; m.p. 234-235 °C. ^{1}H NMR (300 MHz, $CDCl_3$, δ ppm): 2.40 (s, 3H), 3.70 (s, 2H), 6.97-6.99 (m, 4H), 7.21 (d, *J* = 7.8 Hz, 2H), 7.26 (s, 1H), 7.40-7.44 (m, 2H), 7.46-7.51 (m, 2H), 7.70 (td, *J* = 7.5 Hz, 1.8 Hz, 1H), 8.30 (dd, *J* = 6.0 Hz, 1.4 Hz, 1H), 8.52 (s, 1H), 9.97 (d, *J* = 12.3 Hz, 1H); ^{13}C NMR (75 MHz, $CDCl_3$, δ ppm): 20.3, 21.4, 113.8, 116.1, 116.4, 116.9, 117.0, 118.4, 123.7, 123.8, 125.1, 125.8, 128.6, 128.8, 133.8, 137.7, 137.8, 137.9, 140.2, 146.1, 156.3, 156.7, 157.1, 160.3, 179.9, 195.1; HRMS (ESI) calcd for $C_{26}H_{19}FNO_3$ [M - H]$^-$ 412.13490; found 412.13545. Anal. Calcd for $C_{26}H_{20}FNO_3$: C, 75.53; H, 4.88; N, 3.39. Found: C, 75.65; H, 4.78; N, 3.47.

(E)*-3-(2-(4-Methylbenzoyl)-3-(3-nitrophenylamino)allyl)-4*H*-chromen-4-one** (**5*n): Isolated yield 0.343 g (78%); Yellow solid; m.p. 255-256 °C. ^{1}H NMR (300 MHz, $CDCl_3$, δ ppm): 2.33 (s, 3H), 3.63 (s, 2H), 7.15 (d, *J* = 6.6 Hz, 2H), 7.27-7.48 (m, 7H), 7.62-7.73 (m, 3H), 8.24 (d, *J* = 7.2 Hz, 1H), 8.43 (s, 1H), 10.34 (d, *J* = 11.1 Hz, 1H); ^{13}C NMR (75 MHz, $CDCl_3$, δ ppm): 20.7, 21.4, 109.9, 115.9, 116.5, 118.3, 121.3, 123.3, 123.5, 125.2, 125.8, 128.7, 128.9, 130.3, 133.9, 137.3, 140.9, 142.9, 144.0, 149.4, 156.5, 156.6, 180.0, 195.4; HRMS (ESI) calcd for $C_{26}H_{19}N_2O_5$ [M - H]$^-$ 439.12940; found 439.12995. Anal. Calcd for $C_{26}H_{20}N_2O_5$: C, 70.90; H, 4.58; N, 6.36. Found: C, 71.03; H, 4.68; N, 6.31.

(E)*-3-(2-(4-Chlorobenzoyl)-3-(phenylamino)allyl)-4*H*-chromen-4-one** (**5*o): Isolated yield 0.357 g (86%); Colorless solid. m.p. 245-246 °C. ^{1}H NMR (300 MHz, $CDCl_3$, δ ppm): 3.71 (s, 2H), 7.00-7.05 (m, 3H), 7.26-7.33 (m, 2H), 7.37-7.40 (m, 2H), 7.44-7.55 (m, 5H), 7.67-7.72 (m, 1H), 8.31 (d, *J* = 7.8 Hz, 1H), 8.50 (s, 1H), 10.08 (d, *J* = 12.3 Hz, 1H); ^{13}C NMR (75 MHz, $CDCl_3$, δ ppm): 20.2, 113.7, 115.9, 118.4, 122.9, 123.7, 125.1, 125.9, 128.4, 129.7, 129.9, 133.8, 136.1, 139.2, 141.1, 146.2, 156.2, 156.7, 179.8, 193.8; HRMS (ESI) calcd for $C_{25}H_{17}ClNO_3$ [M - H]$^-$ 414.08970; found 414.09024. Anal. Calcd for $C_{25}H_{18}ClNO_3$: C, 72.20; H, 4.36; N, 3.37. Found: C, 72.09; H, 4.24; N, 3.41.

***(E)-3-(2-(3-Nitrobenzoyl)-3-(phenylamino)allyl)-4H-chromen-4-one* (*5p*):** Isolated yield 0.375 g (88%); Yellow solid; m.p. 248-249 ^{o}C. ^{1}H NMR (300 MHz, $CDCl_3$, δ ppm): 3.73 (s, 2H), 7.01-7.06 (m, 3H), 7.26-7.33 (m, 2H), 7.43-7.48 (m, 2H), 7.52 (d, *J* = 8.4 Hz, 1H), 7.60 (t, *J* = 8.0 Hz, 1H), 7.71 (td, *J* = 7.5 Hz, 1.5 Hz, 1H), 7.82 (d, *J* = 7.8 Hz, 1H), 8.29-8.36 (m, 3H), 8.51 (s, 1H), 10.28 (d, *J* = 12.6 Hz, 1H); ^{13}C NMR (75 MHz, $CDCl_3$, δ ppm): 20.2, 113.5, 116.1, 118.4, 123.2, 123.3, 123.5, 123.6, 124.5, 125.2, 125.9, 129.3, 129.7, 133.9, 134.1, 140.8, 142.4, 146.9, 148.1, 156.2, 156.7, 179.9, 192.0; HRMS (ESI) calcd for $C_{25}H_{17}N_2O_5$ $[M - H]^-$ 425.11375; found 425.11430. Anal. Calcd for $C_{25}H_{18}N_2O_5$: C, 70.42; H, 4.25; N, 6.57. Found: C, 70.34; H, 4.31; N, 6.53.

General procedure for the isolation of intermediates 4. A mixture of the suitable enaminone **2 (**1 mmol) and aniline **3** (1 mmol) in DMF (5 mL) was heated at 130 °C for 3 h. The reaction progress was monitored by thin-layer chromatography. After completion of the reaction, the solvent was removed and the product was purified by column chromatography using a petroleum ether–ethyl acetate mixture (4:1 v/v) as eluent.

***(E)-3-(4-methoxyphenylamino)-1-(3-nitrophenyl)prop-2-en-1-one*:** Isolated yield 0.280 g (94%); Orange solid; m.p. 143-144 ^{o}C. ^{1}H NMR (300 MHz, $CDCl_3$, δ ppm): 3.80 (s, 3H), 5.98 (d, *J* = 7.5 Hz, 1H), 6.90 (d, *J* = 8.70 Hz, 2H), 7.08 (d, *J* = 8.70 Hz, 2H), 7.49-7.55 (m, 1H), 7.59-7.65 (m, 1H), 8.22-8.33 (m, 2H), 8.72 (s, 1H), 12.27 (d, *J* = 12.2 Hz, NH); ^{13}C NMR (75 MHz, $CDCl_3$, δ ppm): 55.5, 92.3, 115.1, 118.3, 122.1, 125.5, 129.4, 132.8, 133.2, 140.9, 147.2, 148.4, 156.9, 187.1; Anal. Calcd for $C_{16}H_{14}N_2O_4$: C, 64.42; H, 4.73; N, 9.39. Found: C, 64.55; H, 4.80; N, 9.50.

***(E)-1-phenyl-3-(phenylamino)prop-2-en-1-one*:** Isolated yield 0.201 g (90%); Yellow solid; m.p. 138-139 ^{o}C. ^{1}H NMR (300 MHz, $CDCl_3$, δ ppm): 6.02 (d, *J* = 7.8 Hz, 1H), 7.04-7.10 (m, 3H), 7.30-7.36 (m, 2H), 7.41-7.47 (m, 2H), 7.49-7.54 (m, 2H), 7.88-7.95 (m, 2H),

12.15 (d, J = 11.9 Hz, NH); ^{13}C NMR (75 MHz, $CDCl_3$, δ ppm): 93.7, 116.3, 123.6, 127.3, 128.3, 128.4, 129.7, 131.5, 131.6, 139.2, 139.3, 140.2, 140.3, 144.9, 191.0; Anal. Calcd for $C_{15}H_{13}NO$: C, 80.69; H, 5.87; N, 6.27. Found: C, 80.78; H, 5.83; N, 6.16.

2. X-Ray data for compound 5h (CCDC number 894829)

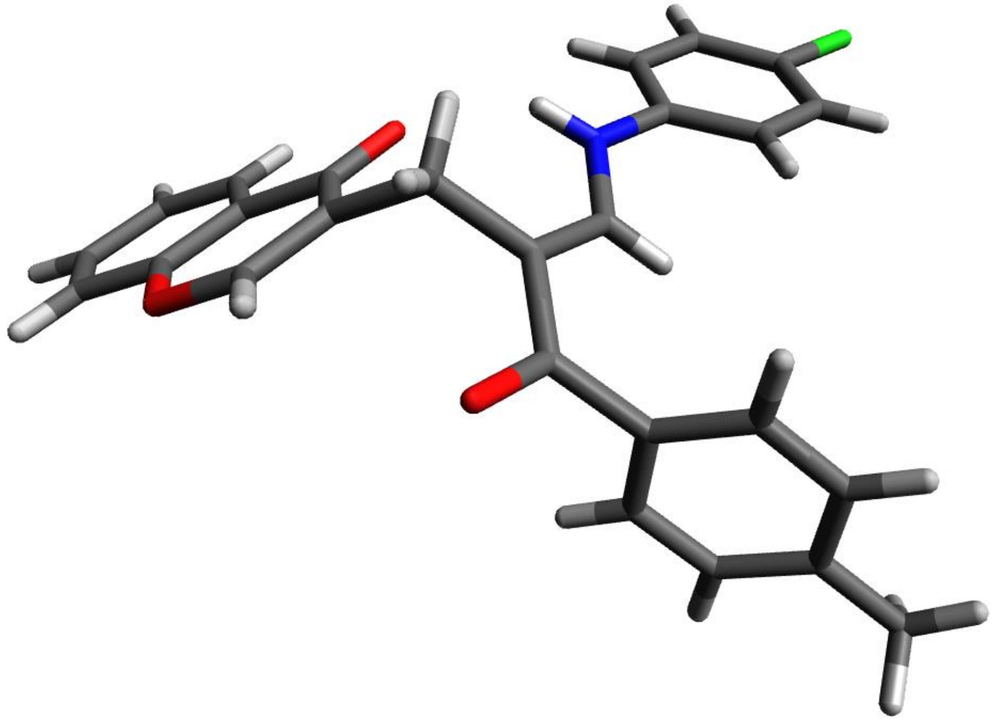

Single Crystal X-Ray structure of compound **5h**

Crystal data of **5h**	
Empirical formula	$C_{26}H_{20}ClN_1O_3$
Formula weight	42988
Temperature	298(2) K
Wavelength	0.71073 Å
Crystal system	Monoclinic
Space group	P 21/n
Unit cell dimensions	a = 13.2215(15) Å α= 90°.<br>b = 7.2887(8) Å β= 94.734(2)°.<br>c = 21.964(3) Å γ= 90°.
Volume	2109.4(4) Å^3
Z	4
Density (calculated)	1.354 kg/m^3
Absorption coefficient	0.210 mm^{-1}
F(000)	896
Crystal size	0.24 x 0.22 x 0.17 mm^3
Theta range for data collection	2.0 to 25.0°.
Index ranges	-17≤h≤17, -9≤k≤9, -28≤l≤28
Reflections collected/ unique	23517/ 5204 [R(int) = 0.0305]
Completeness to theta = 25.0°	100 %

Absorption correction	Psi scan
Refinement method	Full-matrix least-squares on F2
Data / restraints / parameters	5036 / 0 / 280
Goodness-of-fit on F2	1.055
Final R indices [I>2sigma(I)]	R1 = 0.0747, wR2 = 0.0538
R indices (all data)	R1 = 0.1593, wR2 = 0.1454
Largest diff. peak and hole	0.252 and -0.256 e.Å^{-3}

3. NMR assignments of compound 5h

The ^{1}H NMR spectrum of **5h** gives a singlet at 3.70 ppm for the methylenic hydrogens (1-CH_2), which show a C,H-COSY correlation with the carbon signal at 20.4 ppm. These hydrogens also show HMBCs with C-2', C-3', C-4', C-1'', C-2 and C-3 at 156.3, 123.7, 179.9, 195.2, 114.4 and 145.3 ppm respectively. The singlet at 8.51 ppm assignable to H-2' shows (i) a C,H-COSY correlation with the carbon signal at 156.3 ppm and (ii) HMBCs with C-1, C-3' and C-4' at 20.4, 123.7 and 179.9 ppm respectively. The NH hydrogen appears as a doublet at 10.4 ppm (*J* = 12.3 Hz).

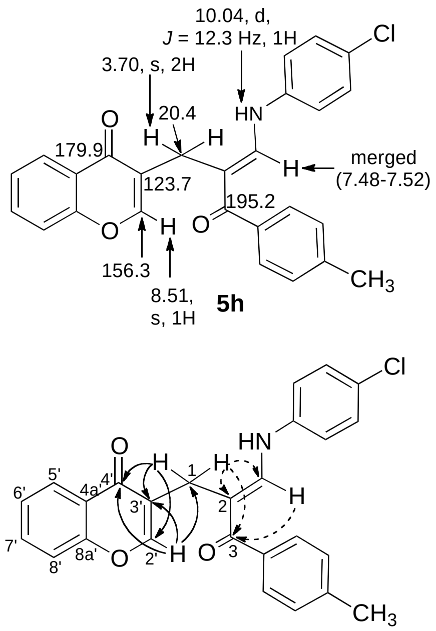

Selected ^{1}H and ^{13}C chemical shifts and HMBC correlations of **5h**

4. Copies of spectra

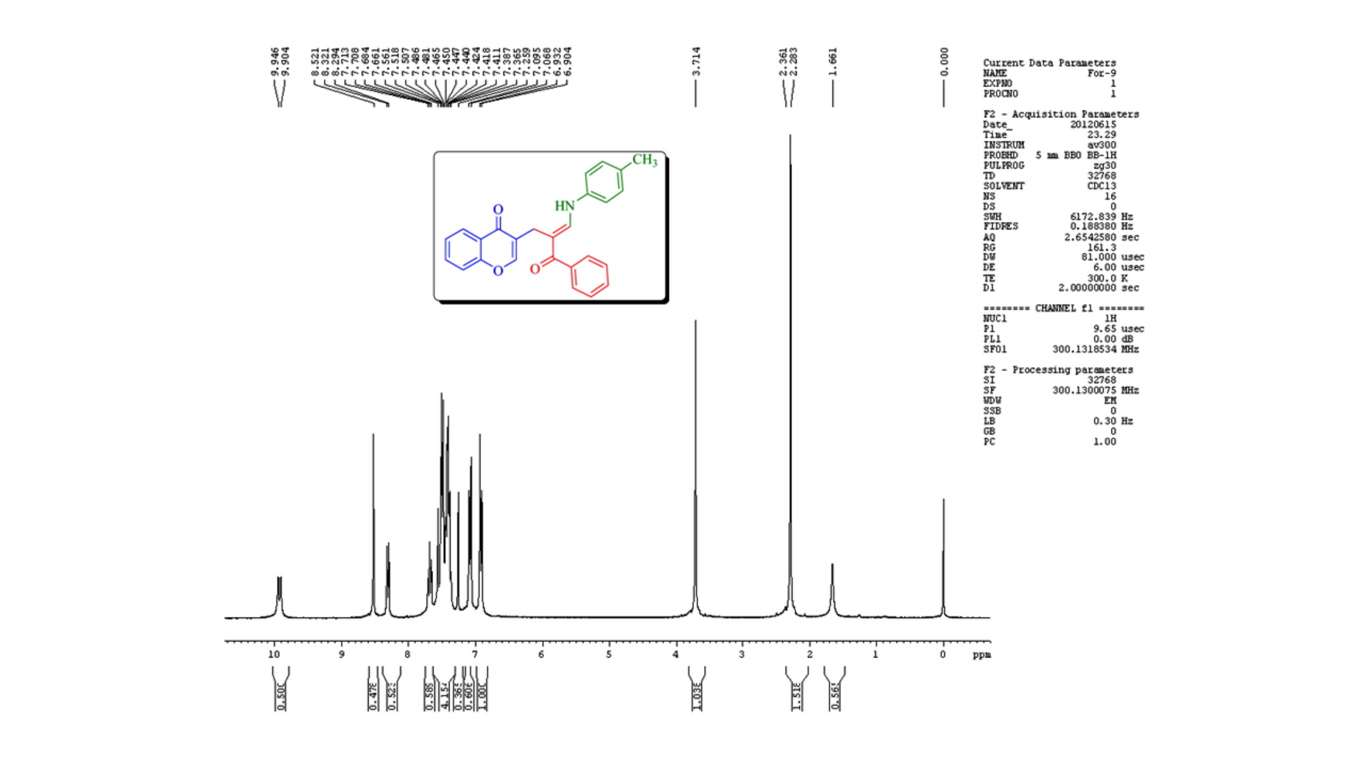

Figure 1. ^{1}H NMR Spectrum of 5a (CDCl$_3$)

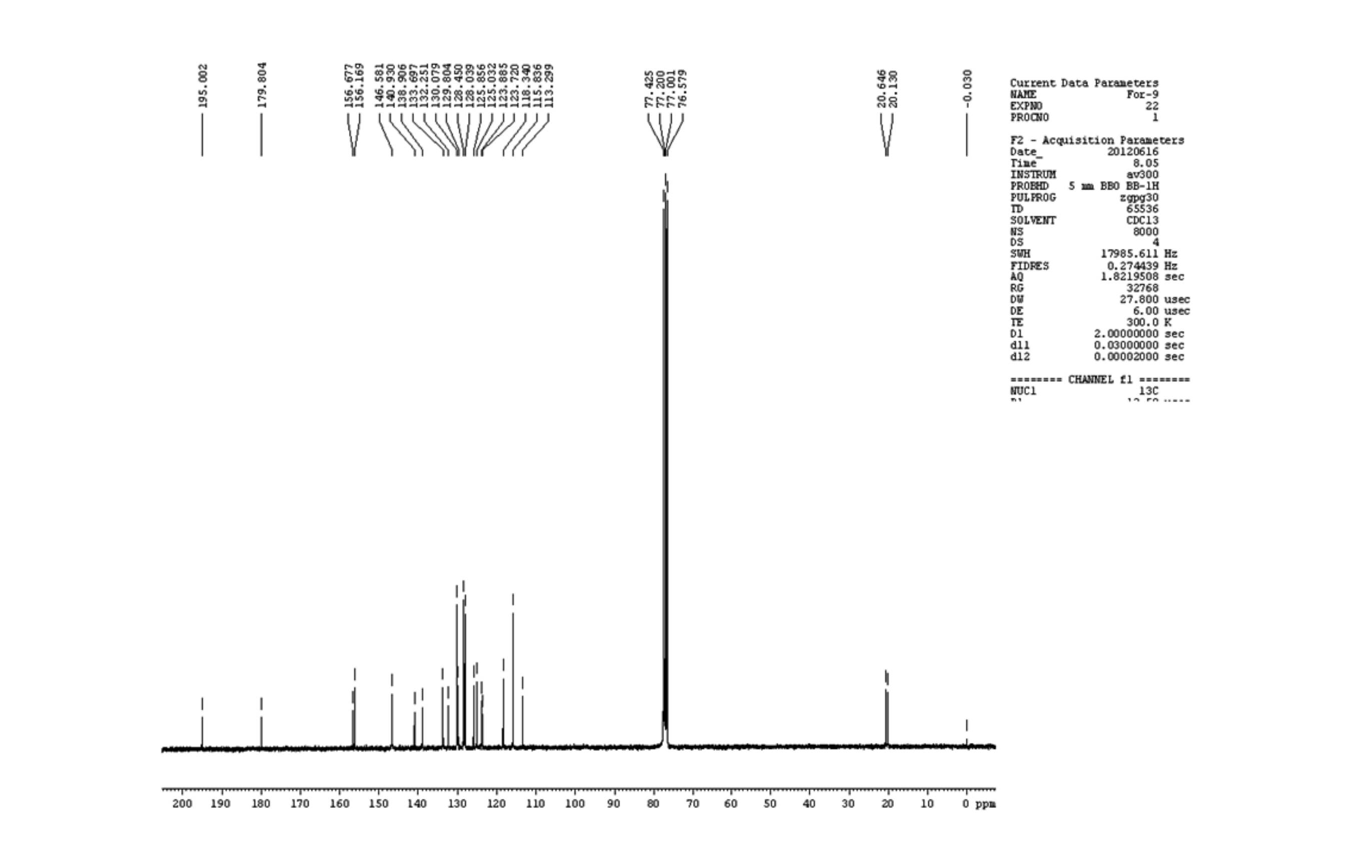

Figure 2. ^{13}C NMR Spectrum of 5a (CDCl$_3$)

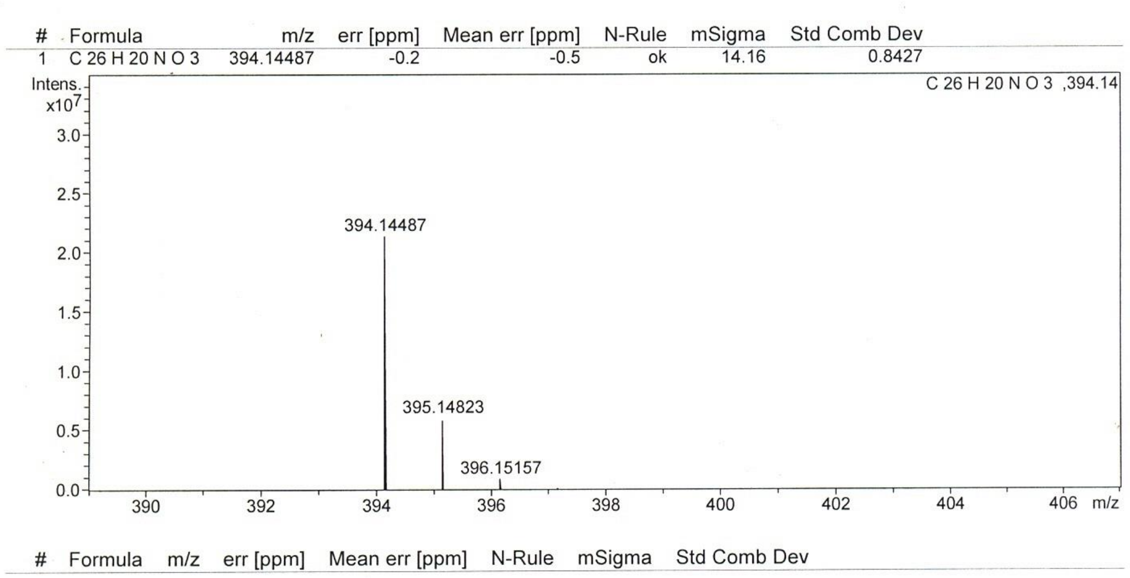

Figure 3. HRMS measurement for **5a**

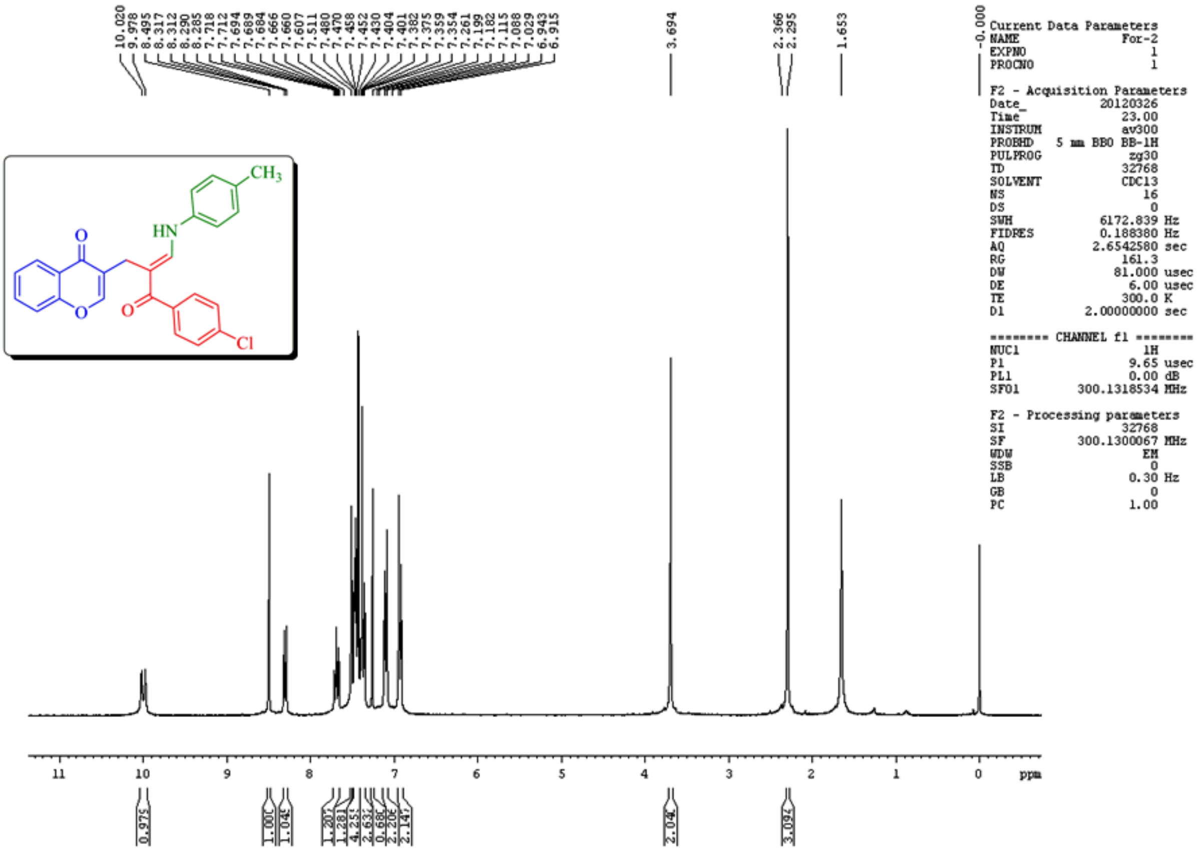

Figure 4. **^{1}H NMR Spectrum of 5b** ($CDCl_3$)

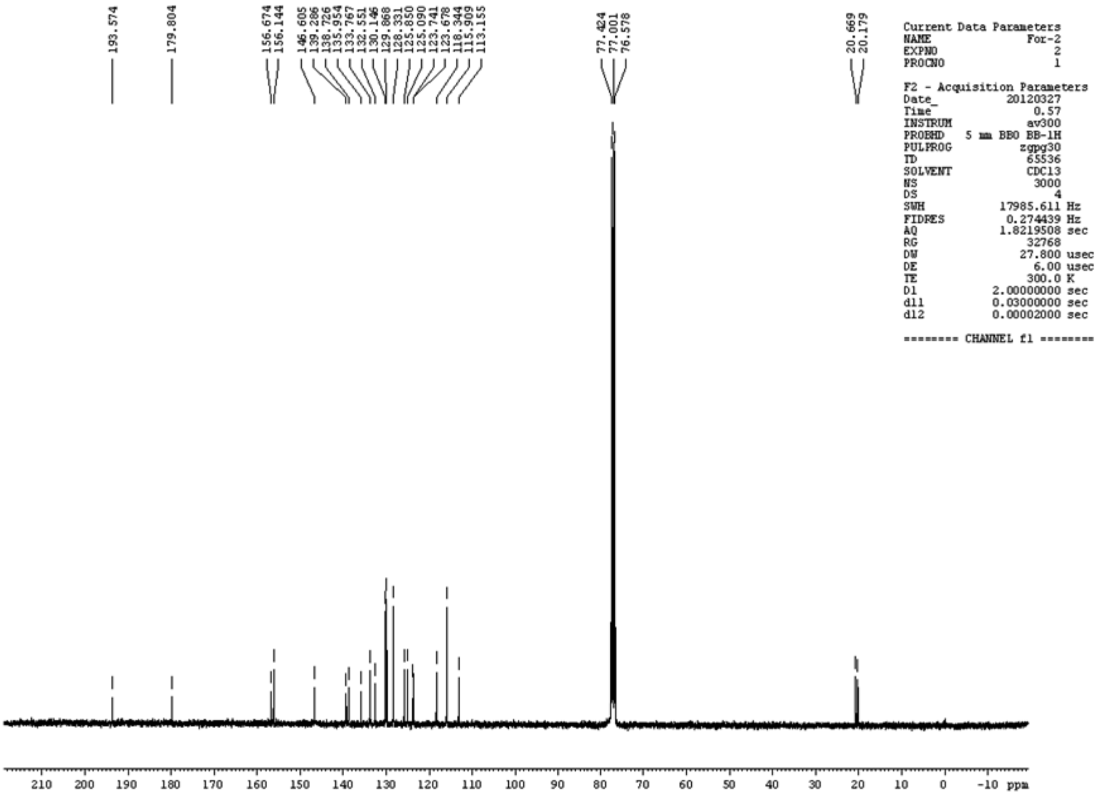

Figure 5. ^{13}**C NMR Spectrum of 5b (**CDCl$_3$)

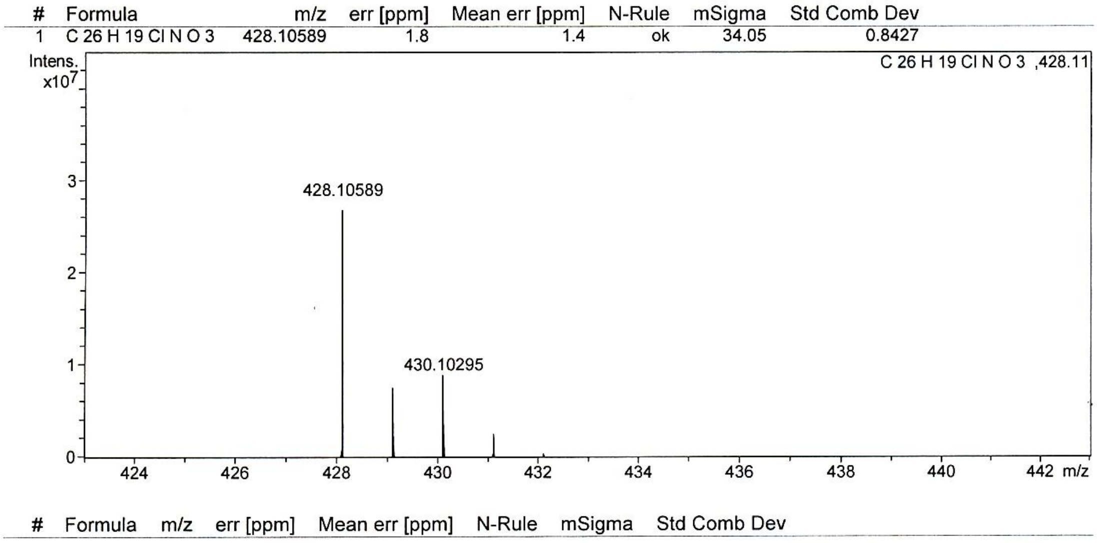

Figure 6. HRMS measurement for **5b**

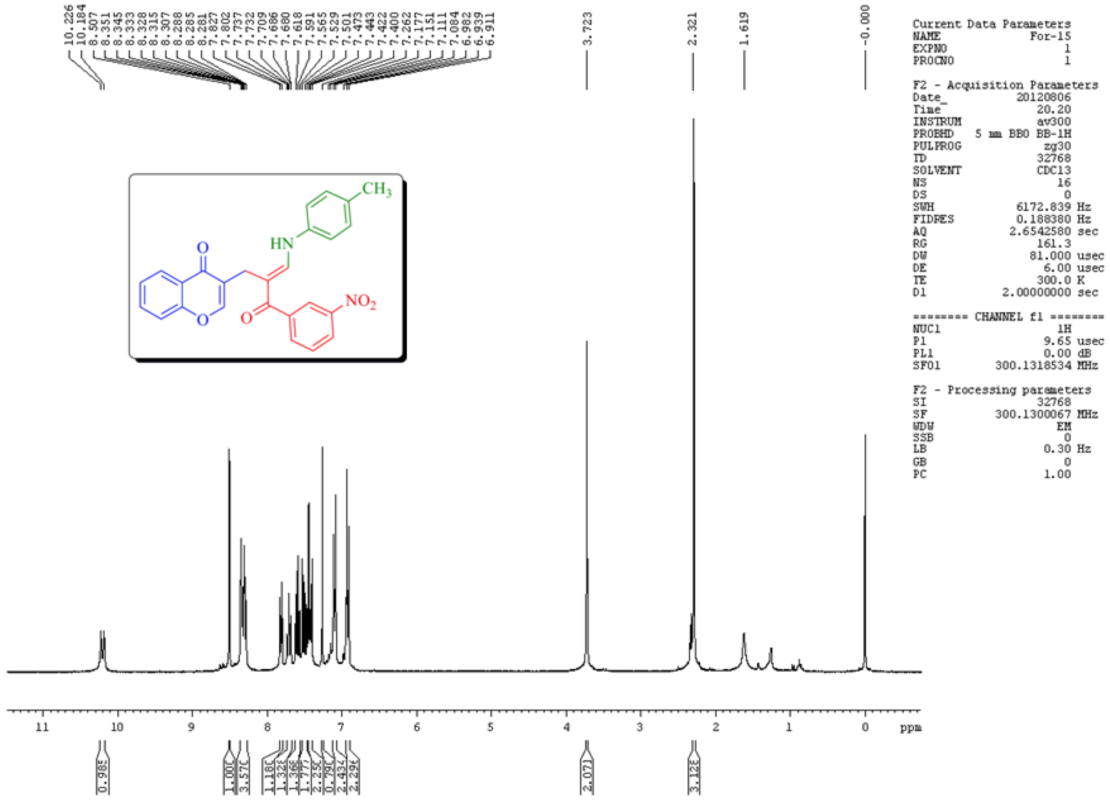

Figure 7. ^{1}H NMR Spectrum of 5c ($CDCl_3$)

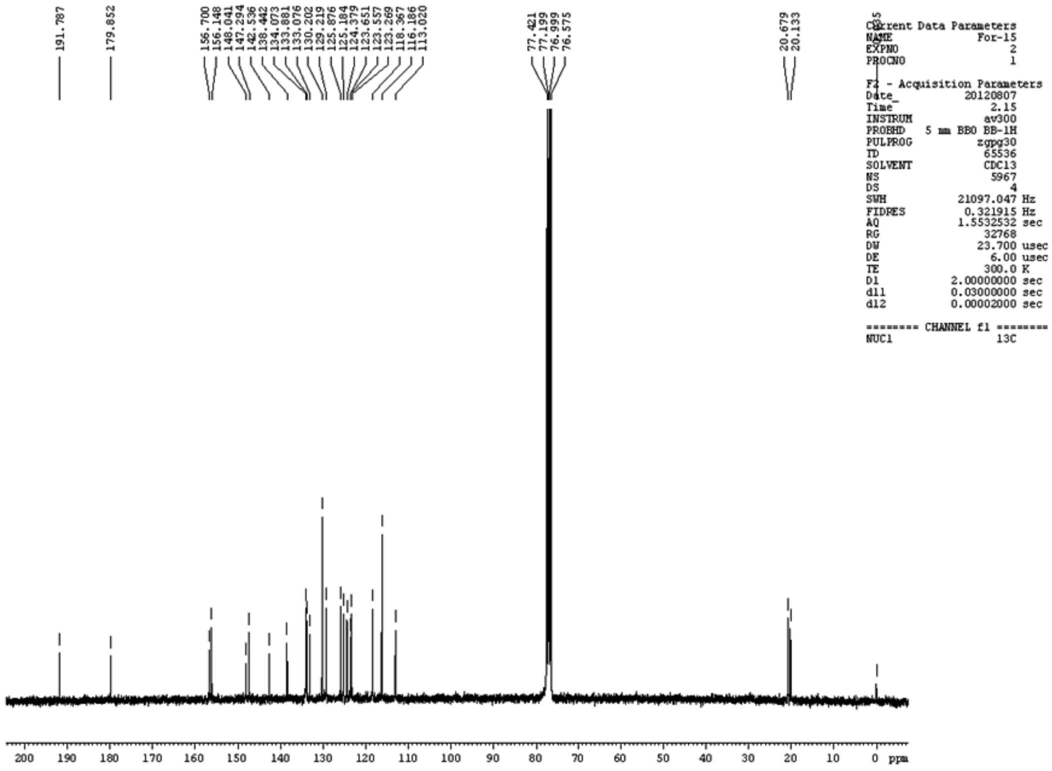

Figure 8. ^{13}C NMR Spectrum of 5c ($CDCl_3$**)**

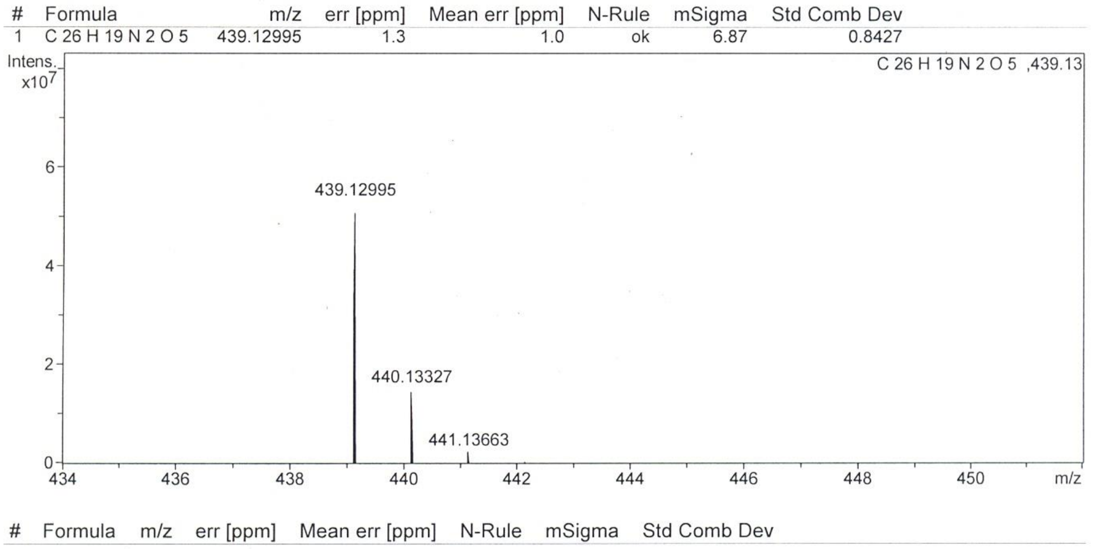

Figure 9. HRMS measurement for **5c**

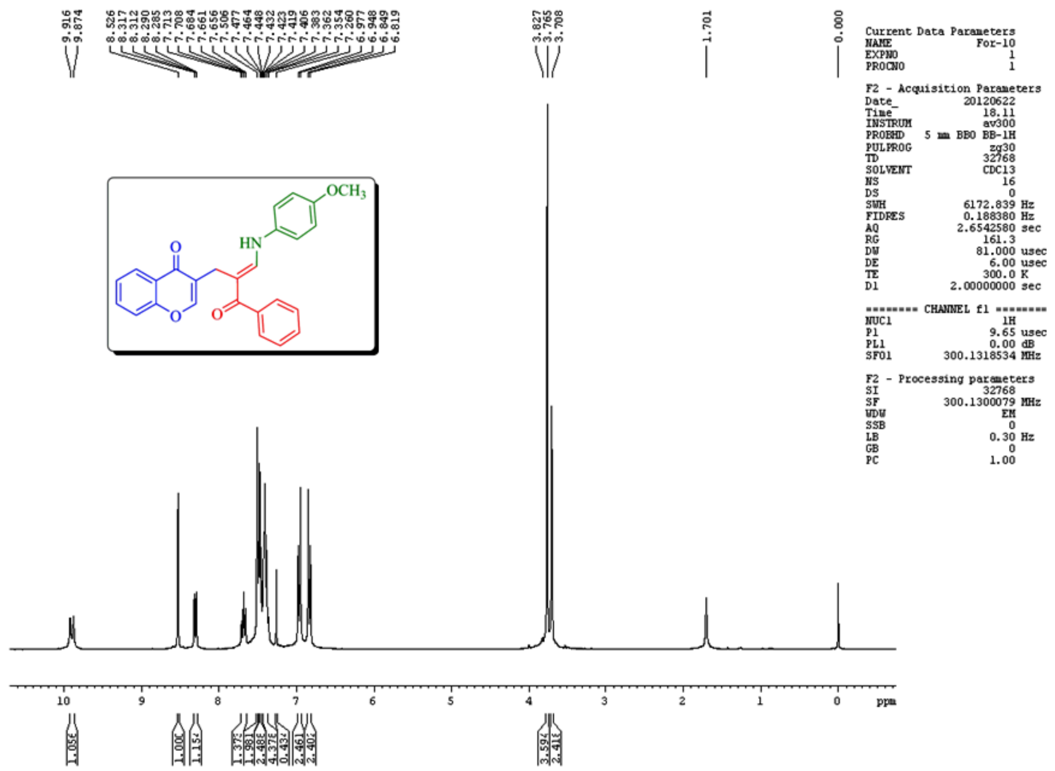

Figure 10. **^{1}H NMR Spectrum of 5d** ($CDCl_3$)

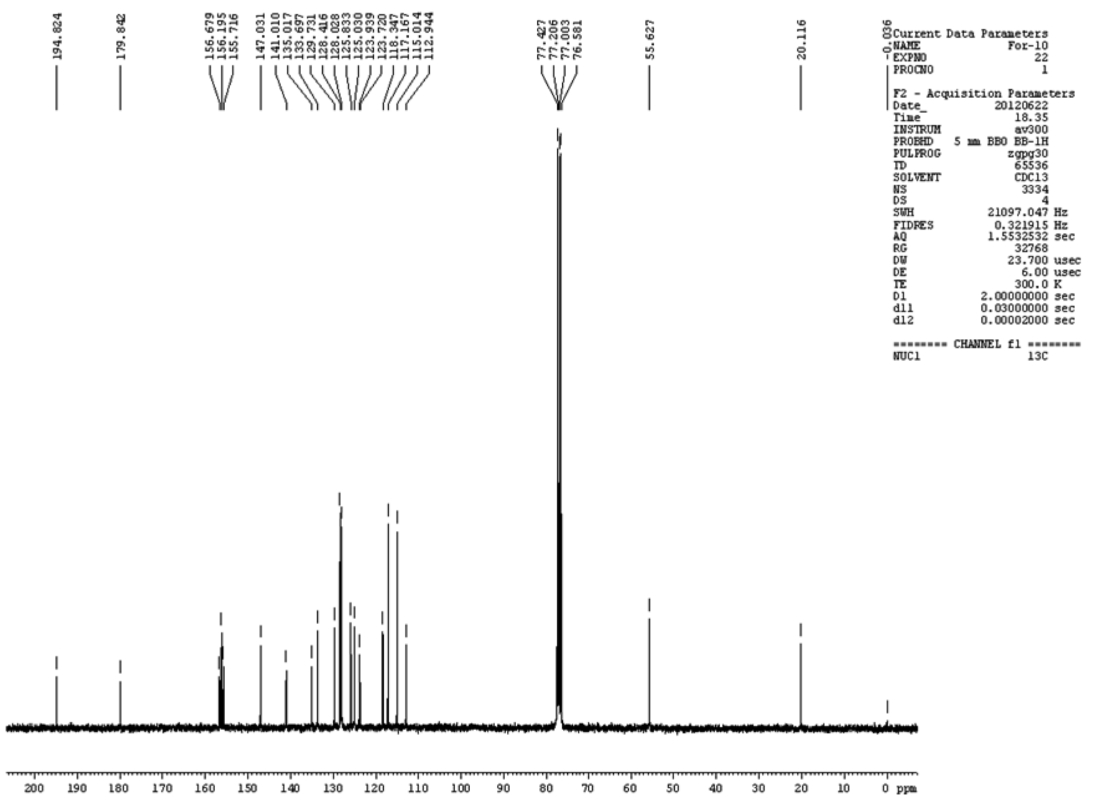

Figure 11. ^{13}C NMR Spectrum of 5d ($CDCl_3$)

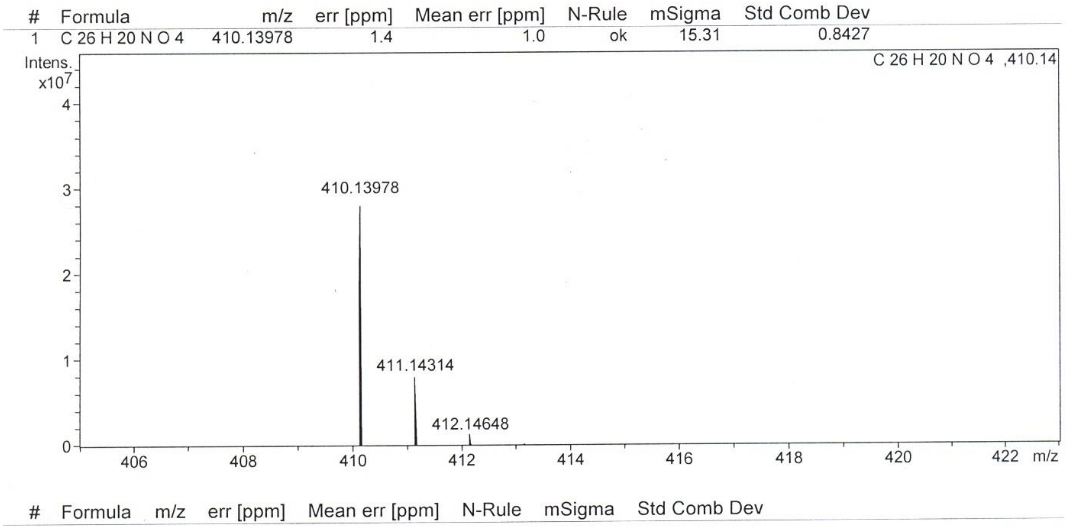

Figure 12. HRMS measurement for **5d**

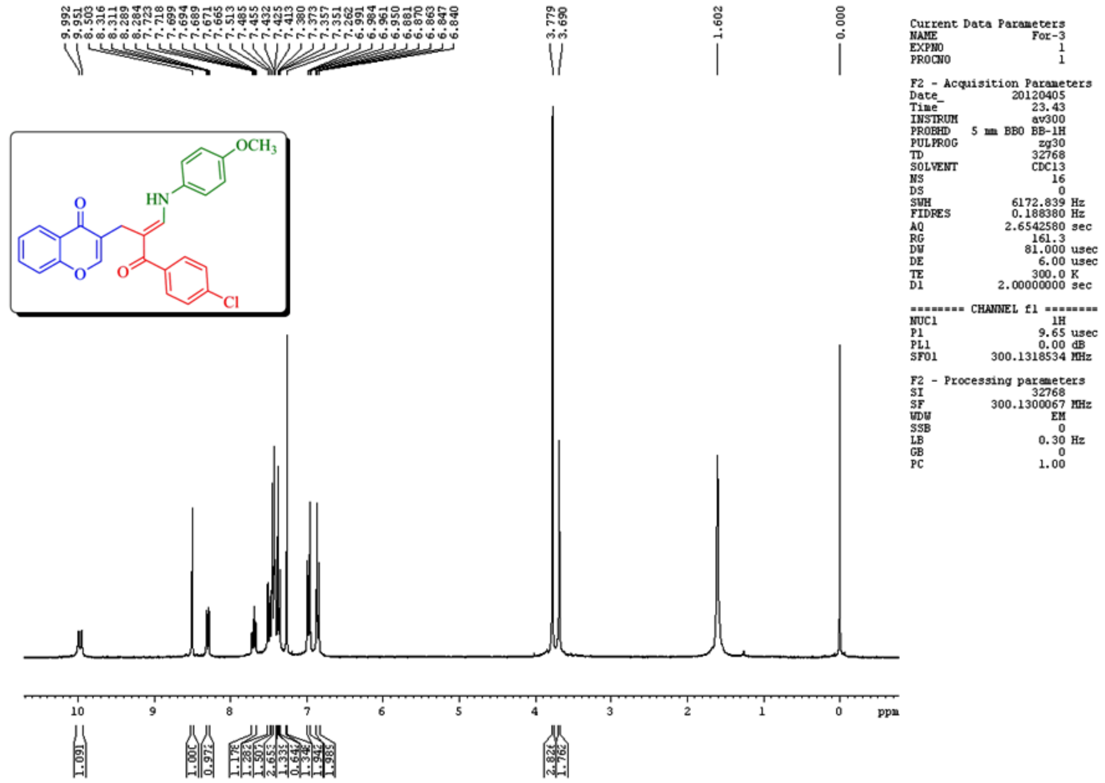

Figure 13. ^{1}H NMR Spectrum of 5e ($CDCl_3$)

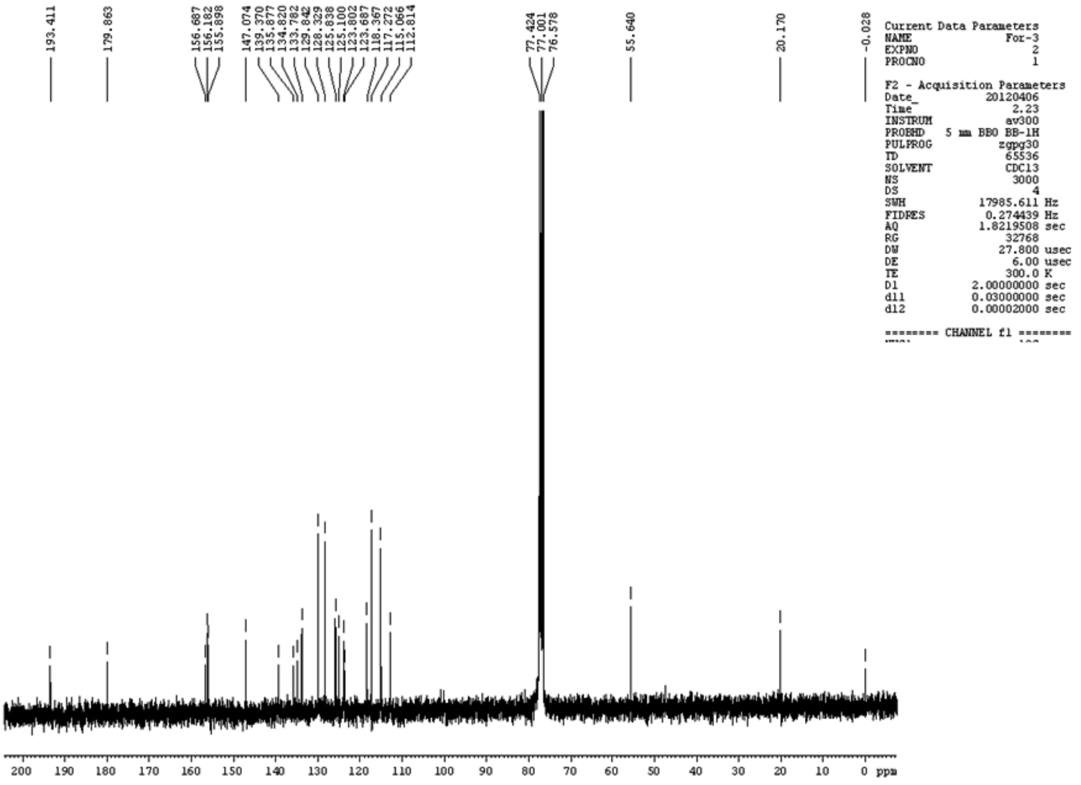

Figure 14. ^{13}C NMR Spectrum of 5e ($CDCl_3$)

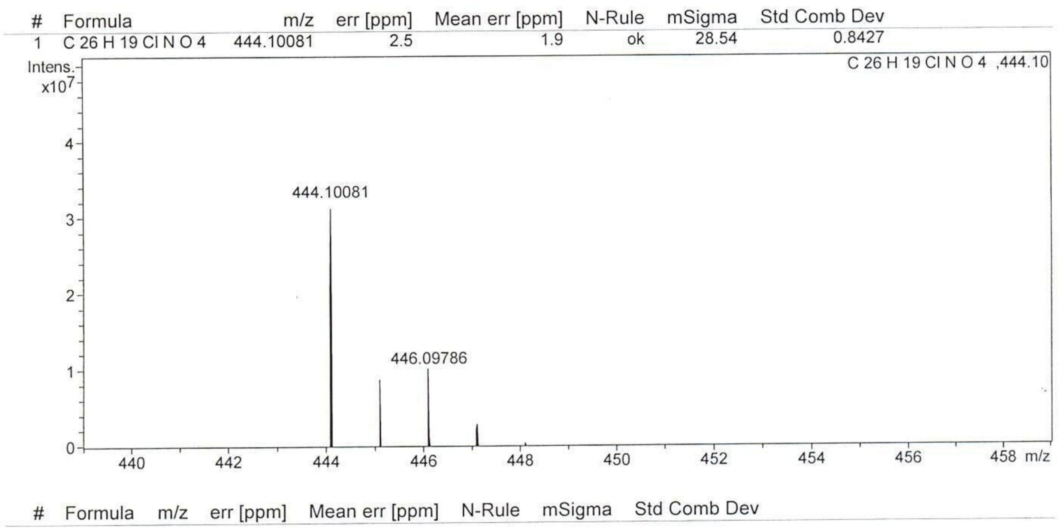

Figure 15. HRMS measurement for **5e**

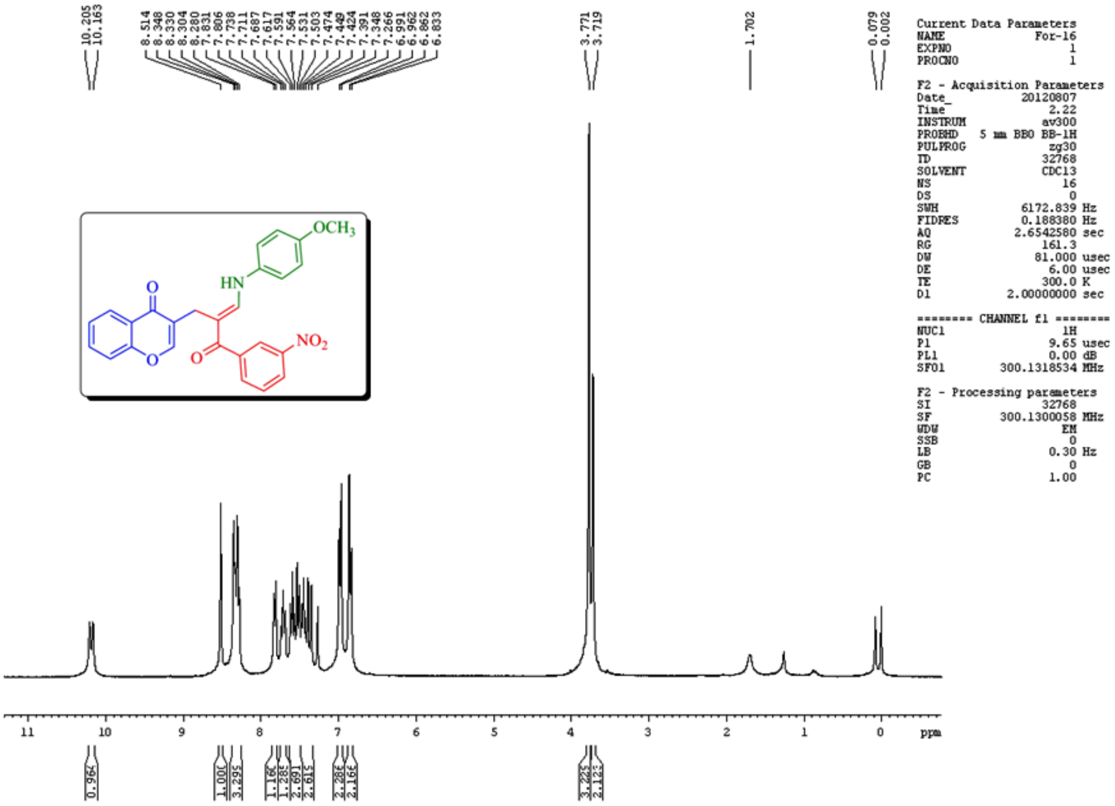

Figure 16. ^{1}H NMR Spectrum of 5f (CDCl$_3$)

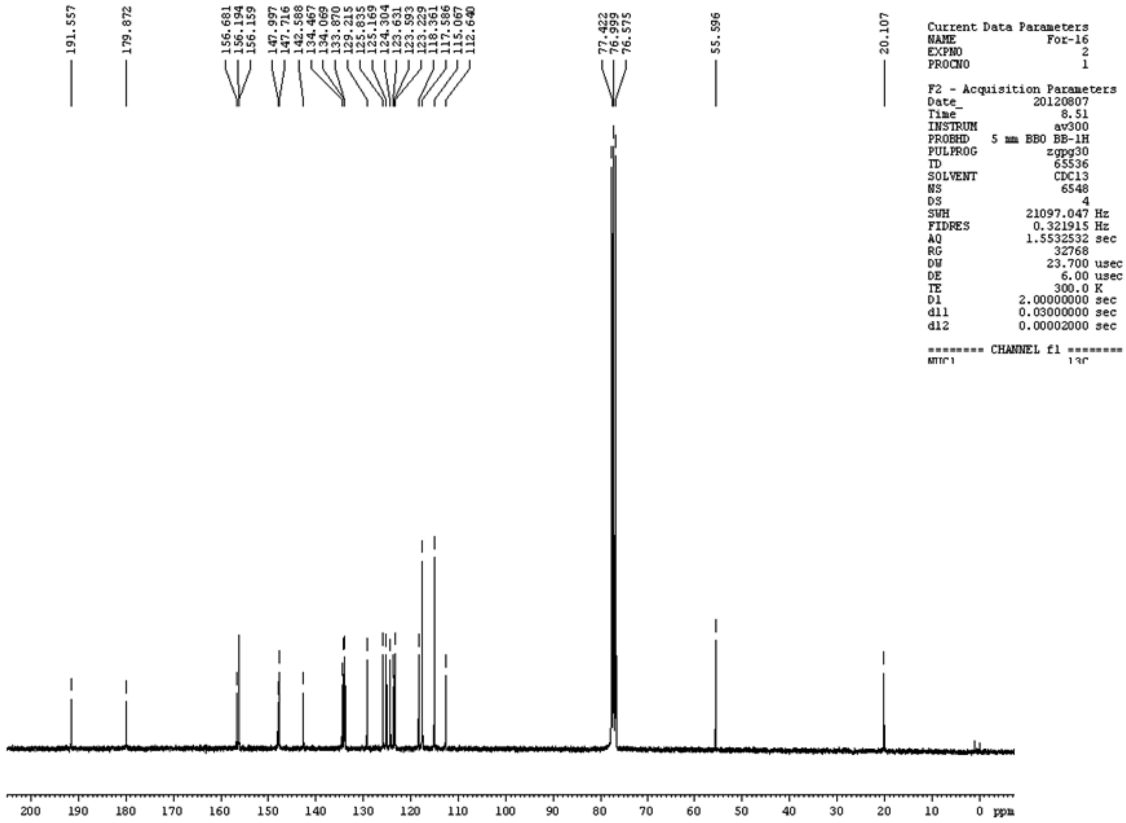

Figure 17. ^{13}C NMR Spectrum of 5f (CDCl$_3$)

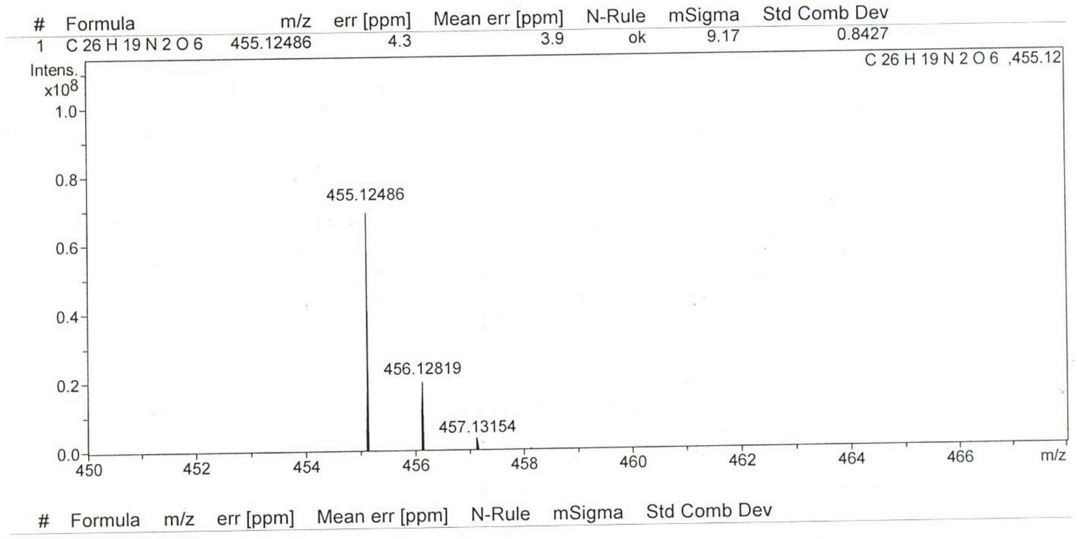

Figure 18.HRMS measurement for **5f**

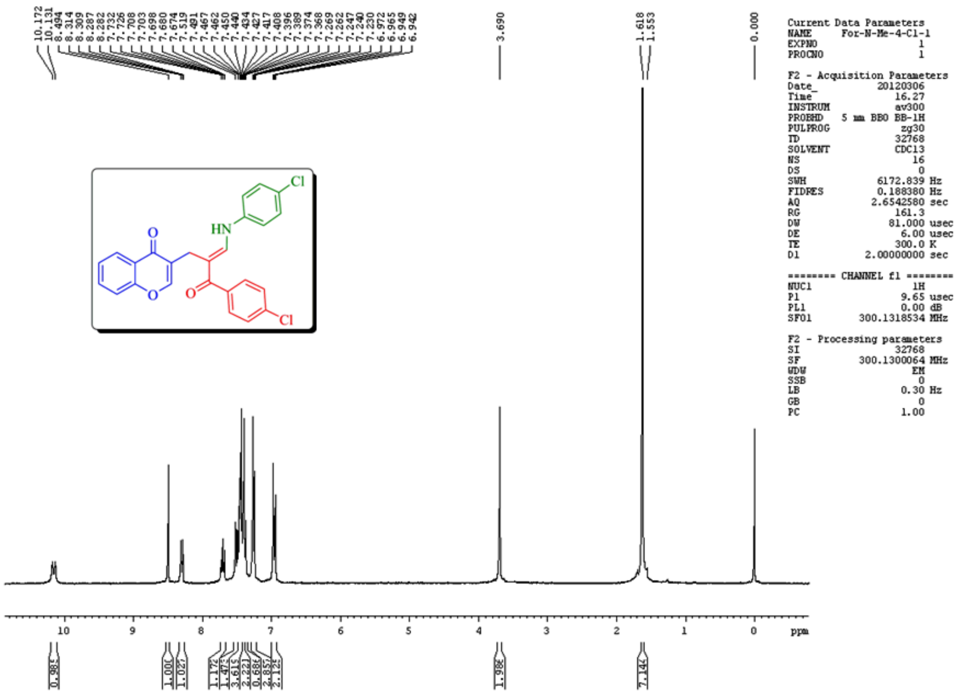

Figure 19. ^{1}**H NMR Spectrum of 5g** ($CDCl_3$)

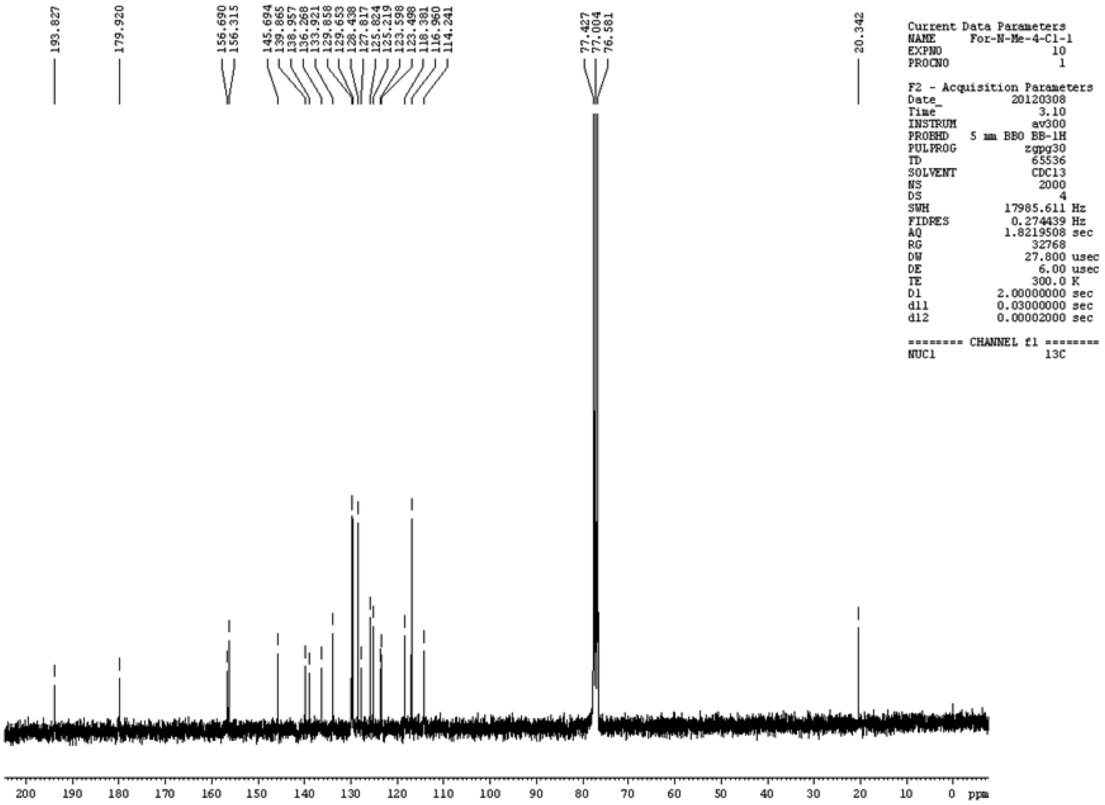

Figure 20. ^{13}**C NMR Spectrum of 5g** ($CDCl_3$)

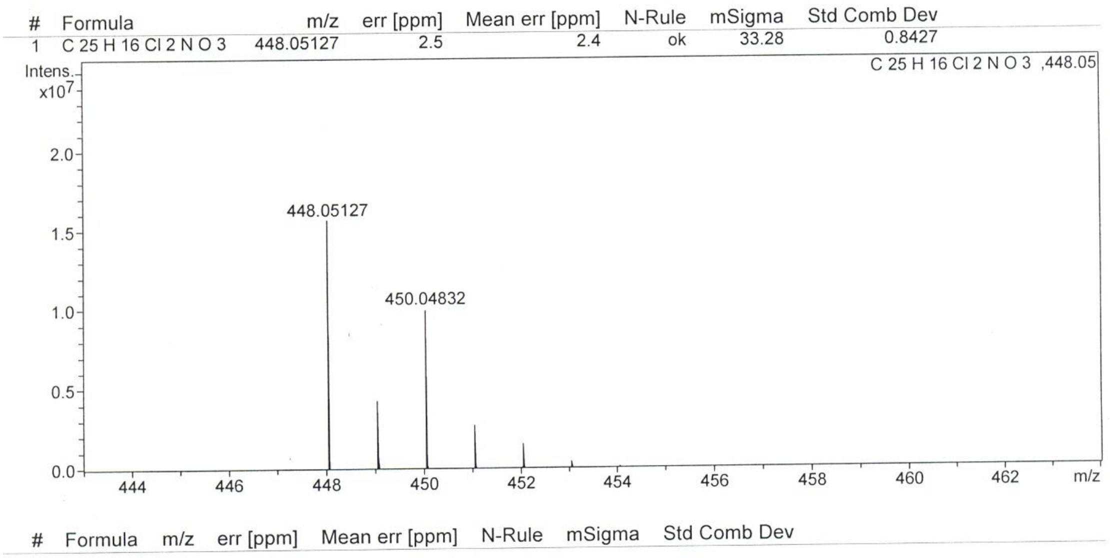

Figure 21. HRMS measurement for **5g**

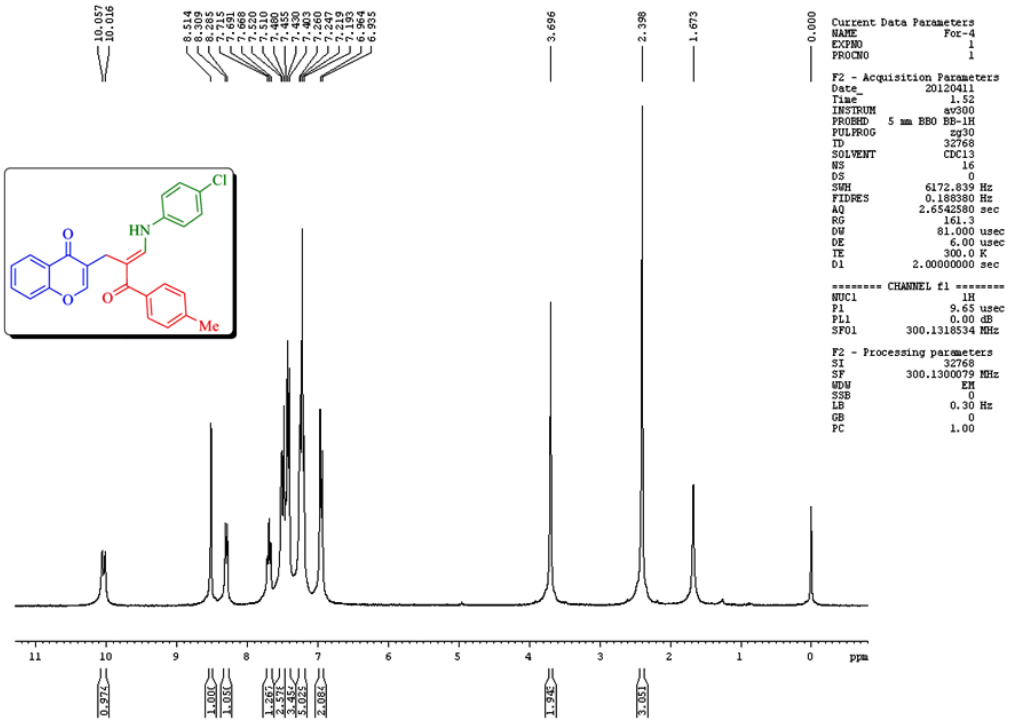

Figure 22. ^{1}**H NMR Spectrum of 5h** ($CDCl_3$)

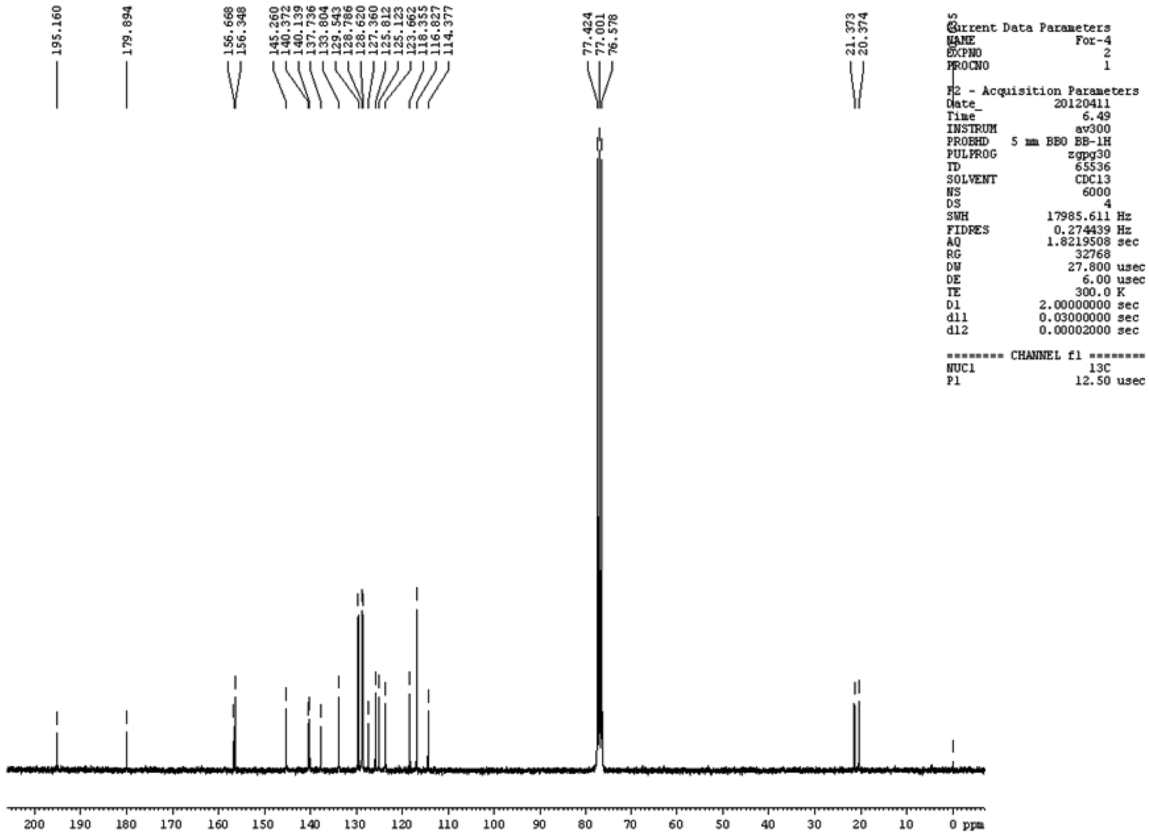

Figure 23. ^{13}**C NMR Spectrum of 5h (**$CDCl_3$)

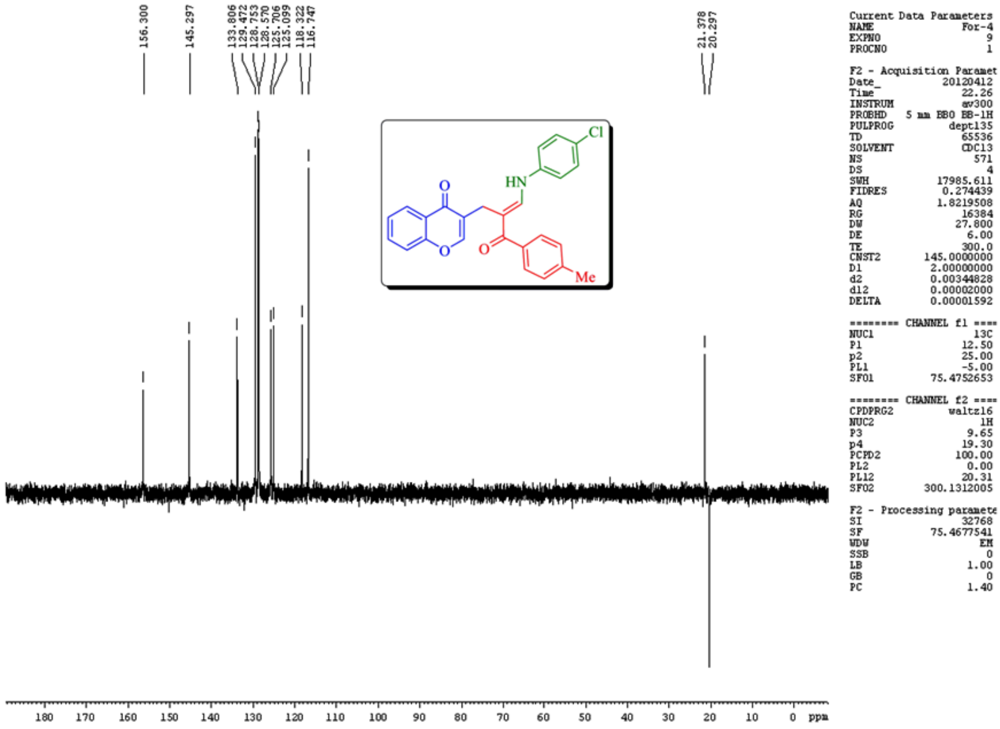

Figure 24. DEPT - 135 NMR Spectrum of 5h ($CDCl_3$)

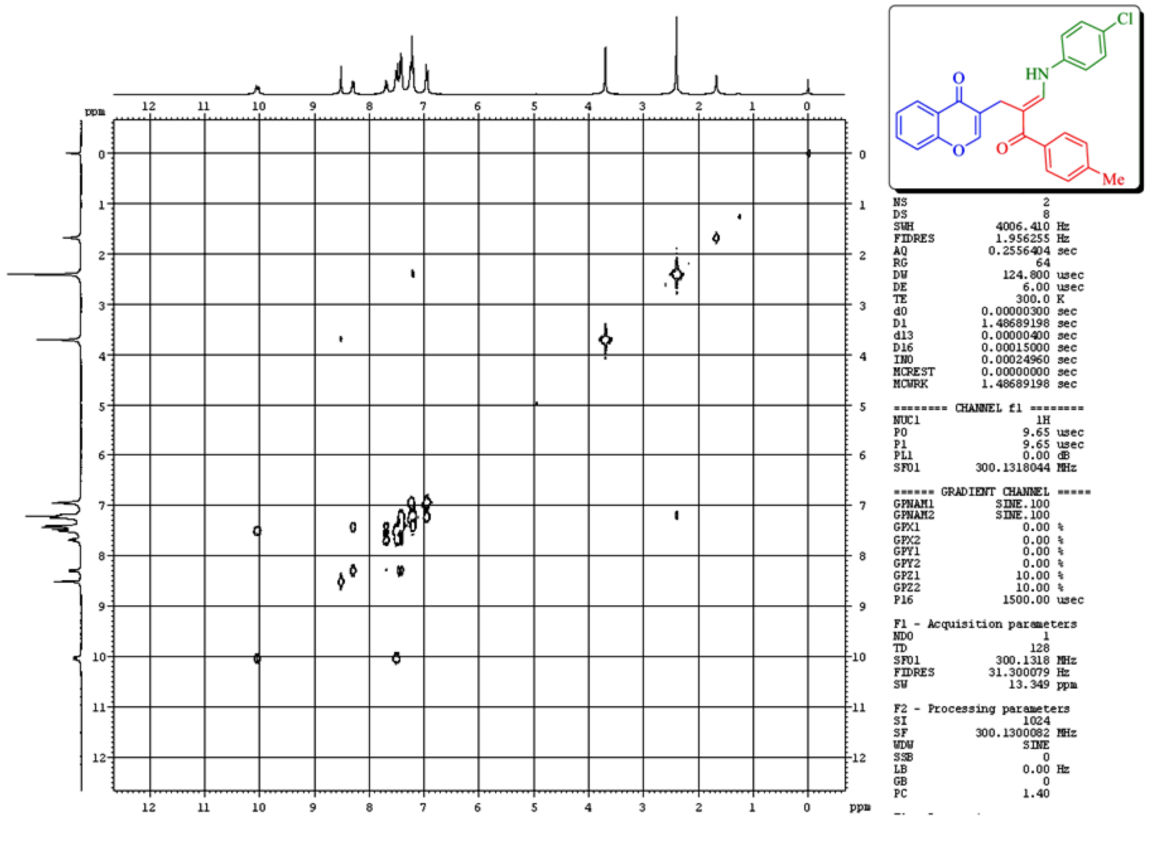

Figure 25. H,H-COSY Spectrum of 5h ($CDCl_3$)

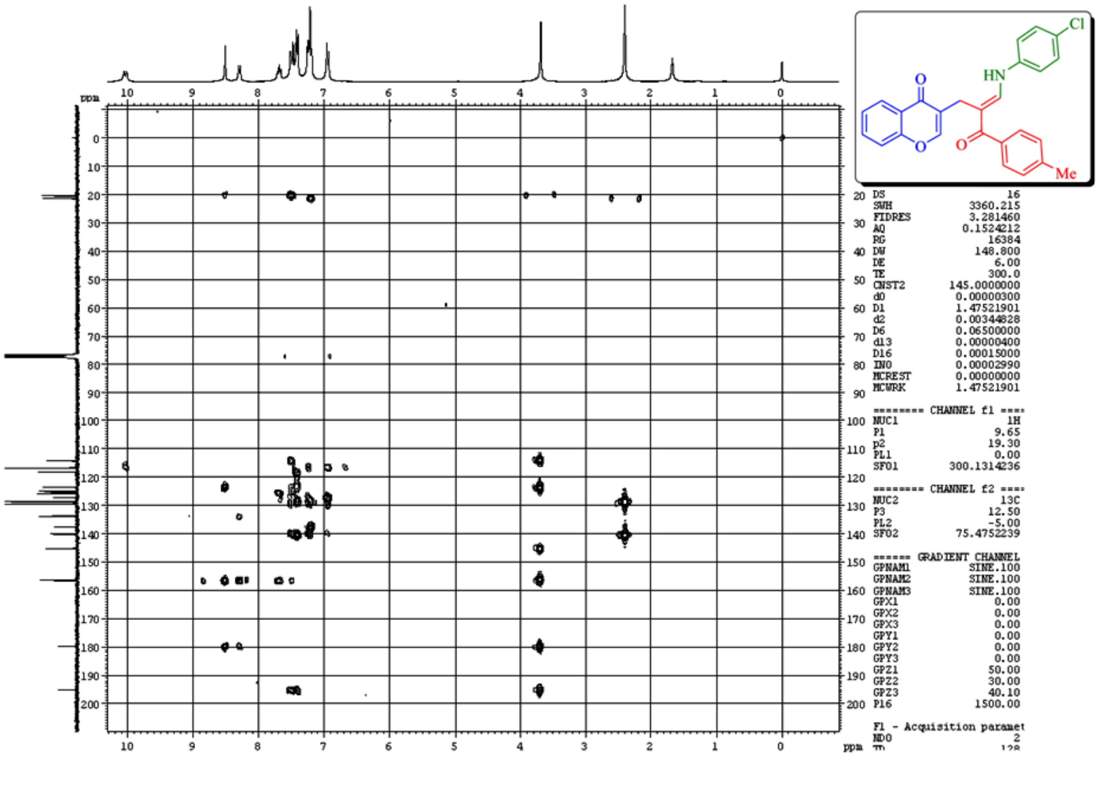

Figure 26. HMBC Spectrum of 5h ($CDCl_3$)

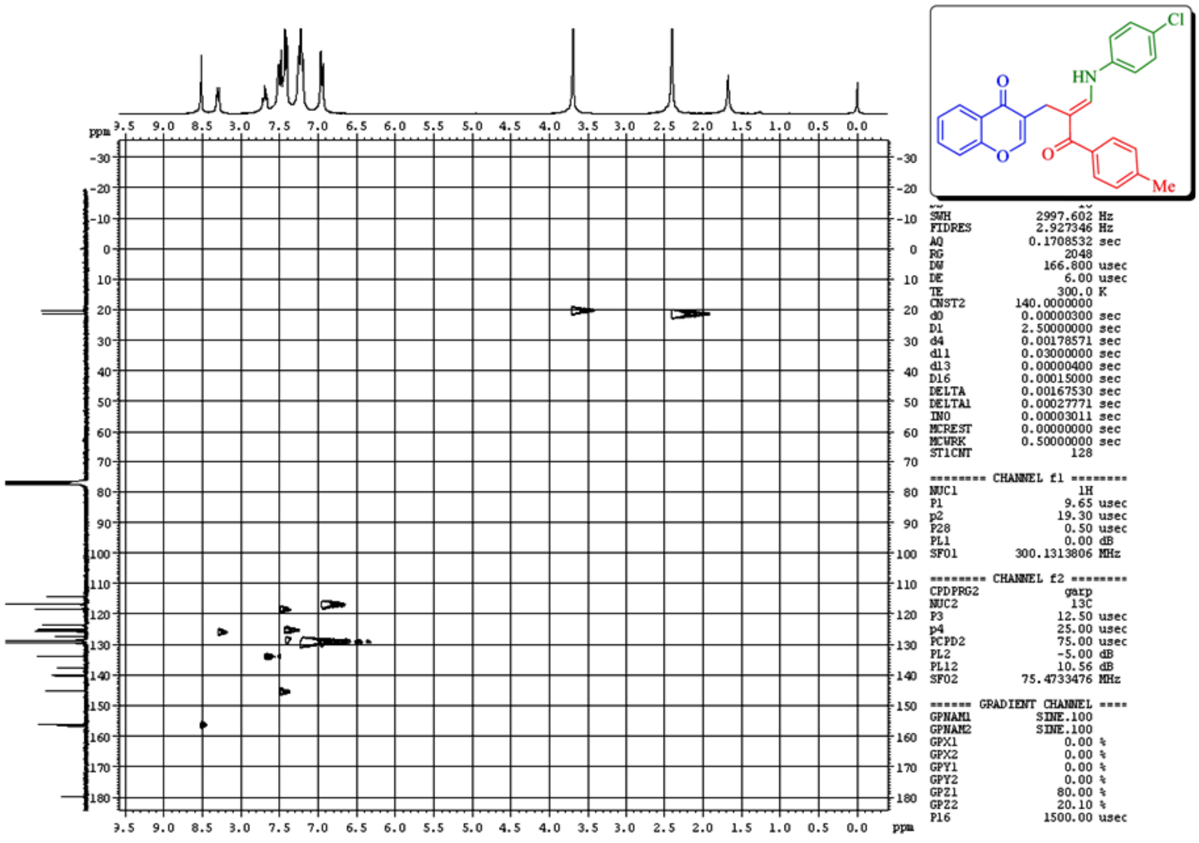

Figure 27. C,H-COSY Spectrum of 5h (CDCl$_3$)

#	Formula	m/z	err [ppm]	Mean err [ppm]	N-Rule	mSigma	Std Comb Dev
1	C 26 H 19 Cl N O 3	428.10589	1.4	0.7	ok	22.36	0.8427

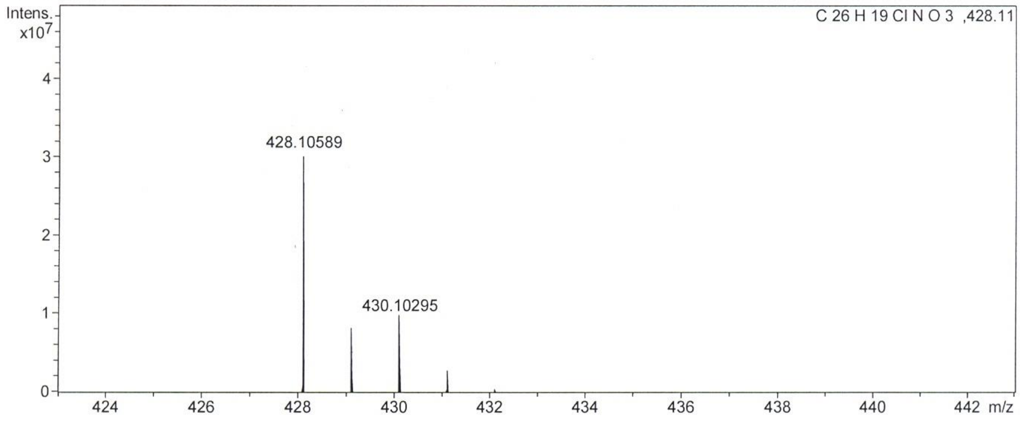

#	Formula	m/z	err [ppm]	Mean err [ppm]	N-Rule	mSigma	Std Comb Dev

Figure 28. HRMS measurement for **5h**

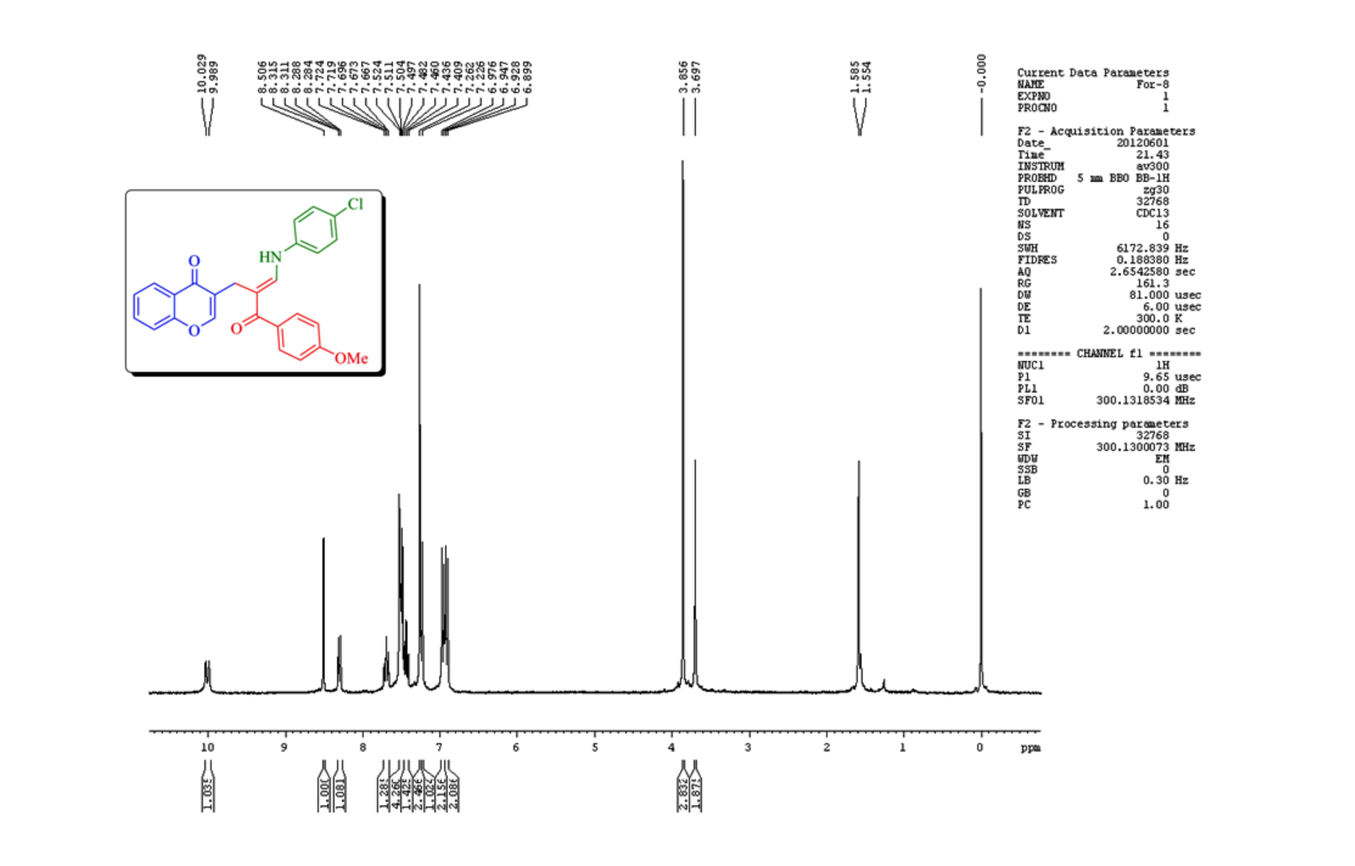

Figure 29. ^{1}**H NMR Spectrum of 5i** ($CDCl_3$)

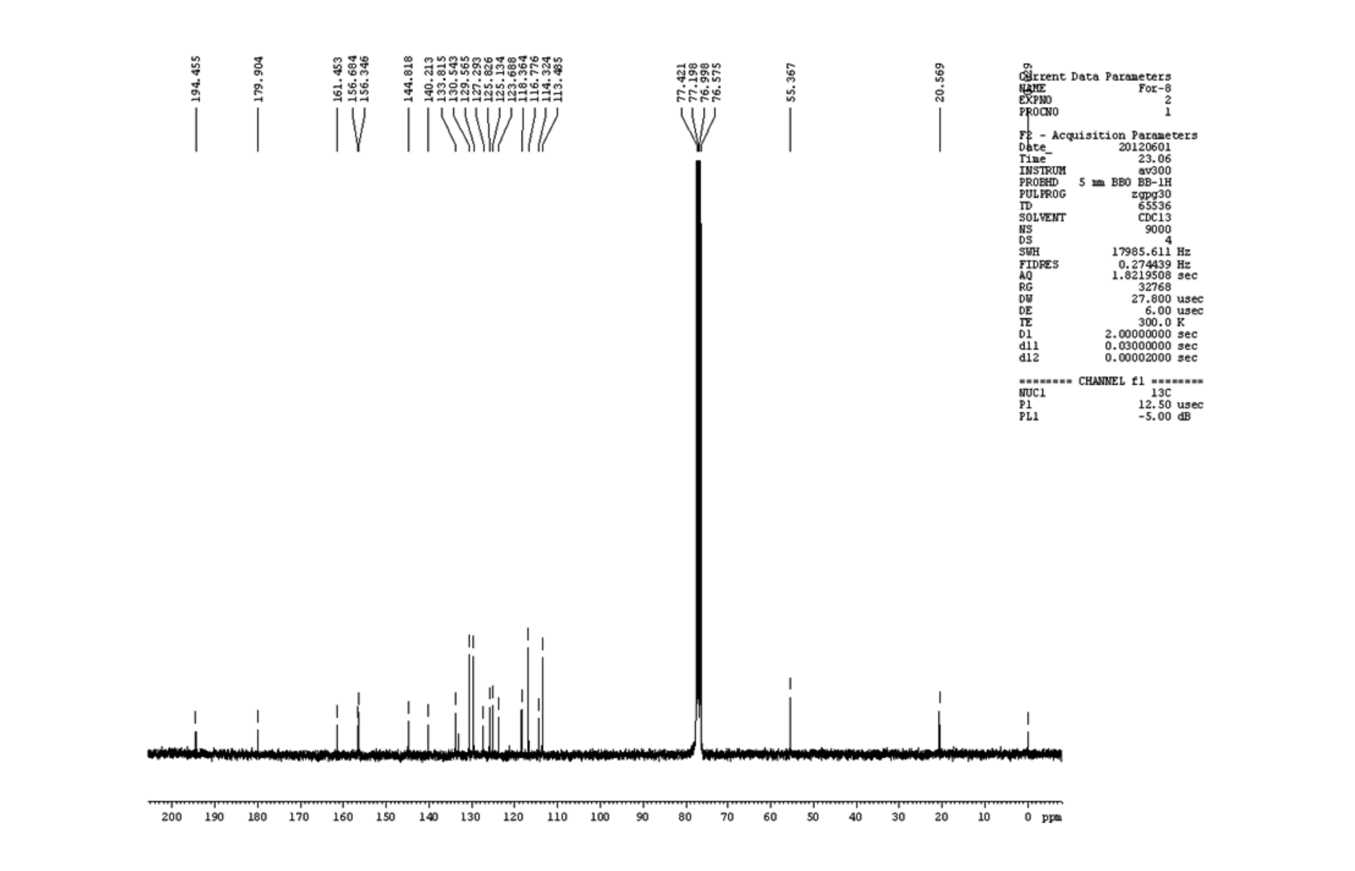

Figure 30. ^{13}**C NMR Spectrum of 5i** ($CDCl_3$)

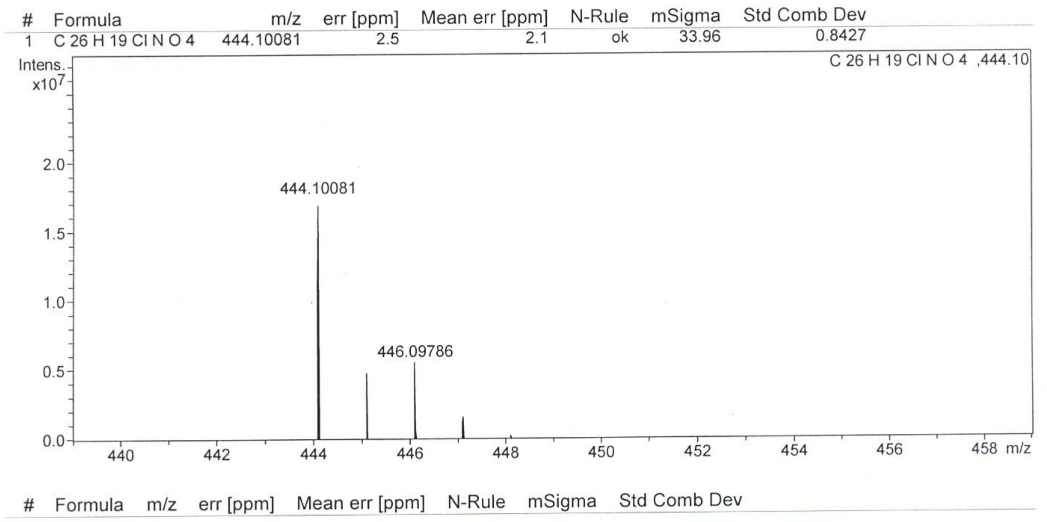

Figure 31. HRMS measurement for **5i**

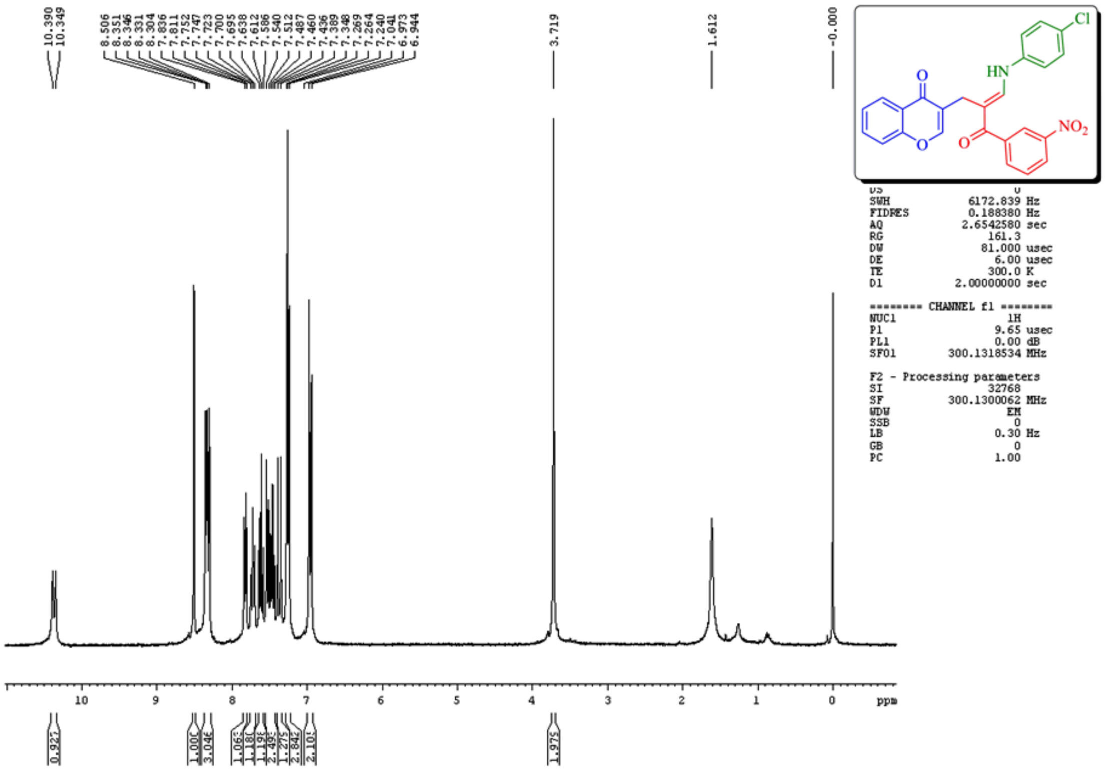

Figure 32. ^{1}**H NMR Spectrum of 5j** ($CDCl_3$)

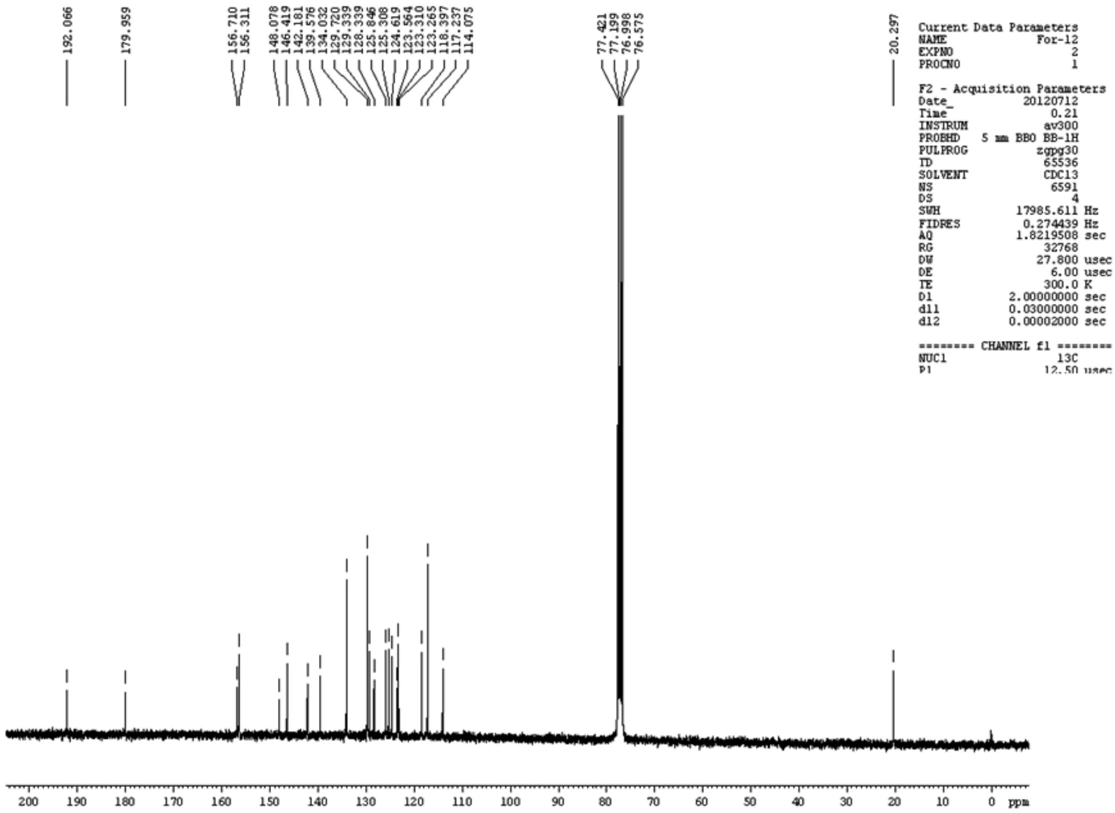

Figure 33. ^{13}**C NMR Spectrum of 5j (**$CDCl_3$)

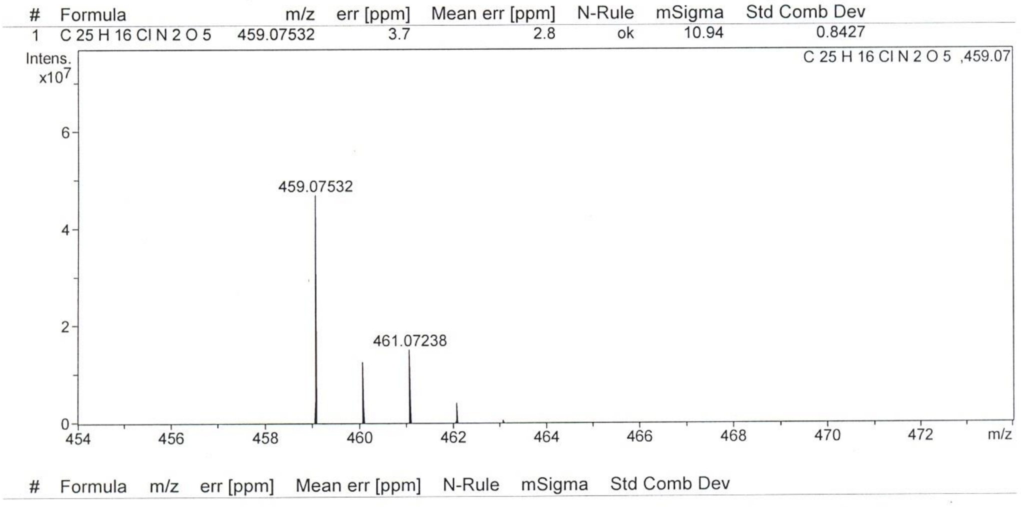

Figure 34. HRMS measurement for **5j**

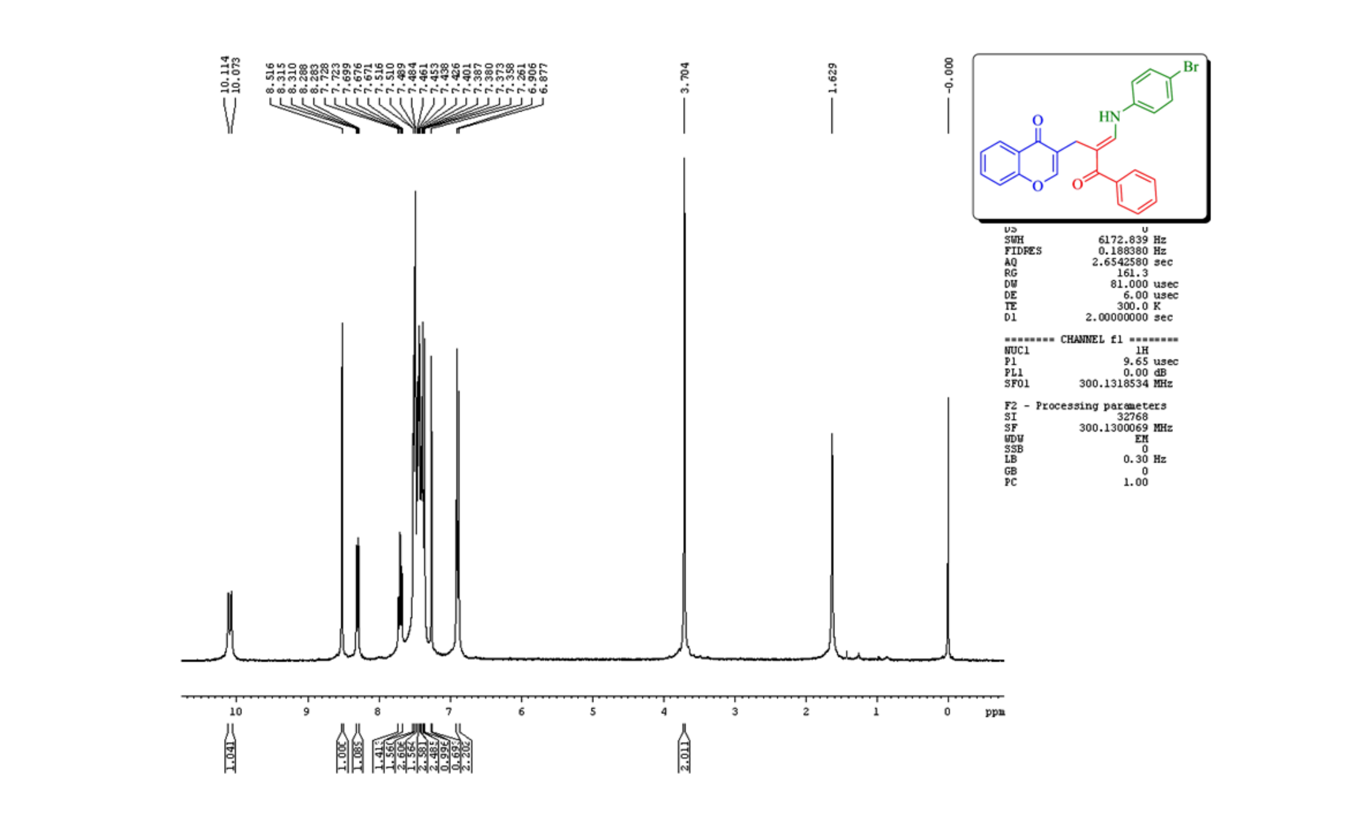

Figure 35. ¹H NMR Spectrum of 5k (CDCl$_3$)

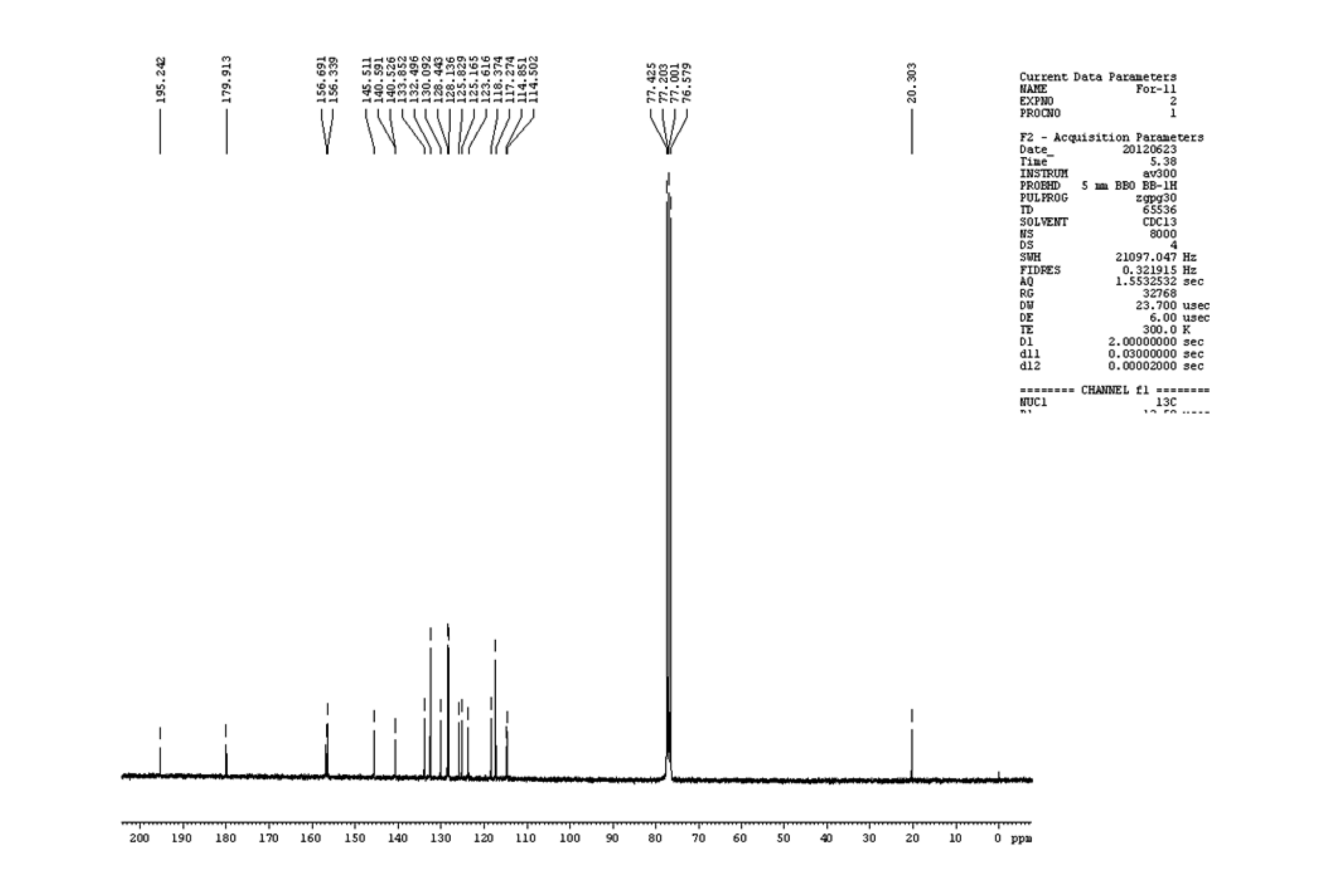

Figure 36. ¹³C NMR Spectrum of 5k (CDCl$_3$)

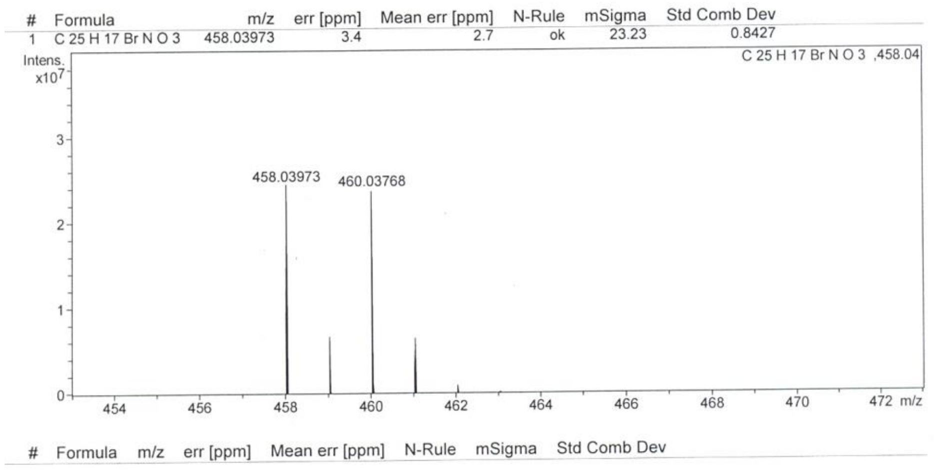

Figure 37. HRMS measurement for **5k**

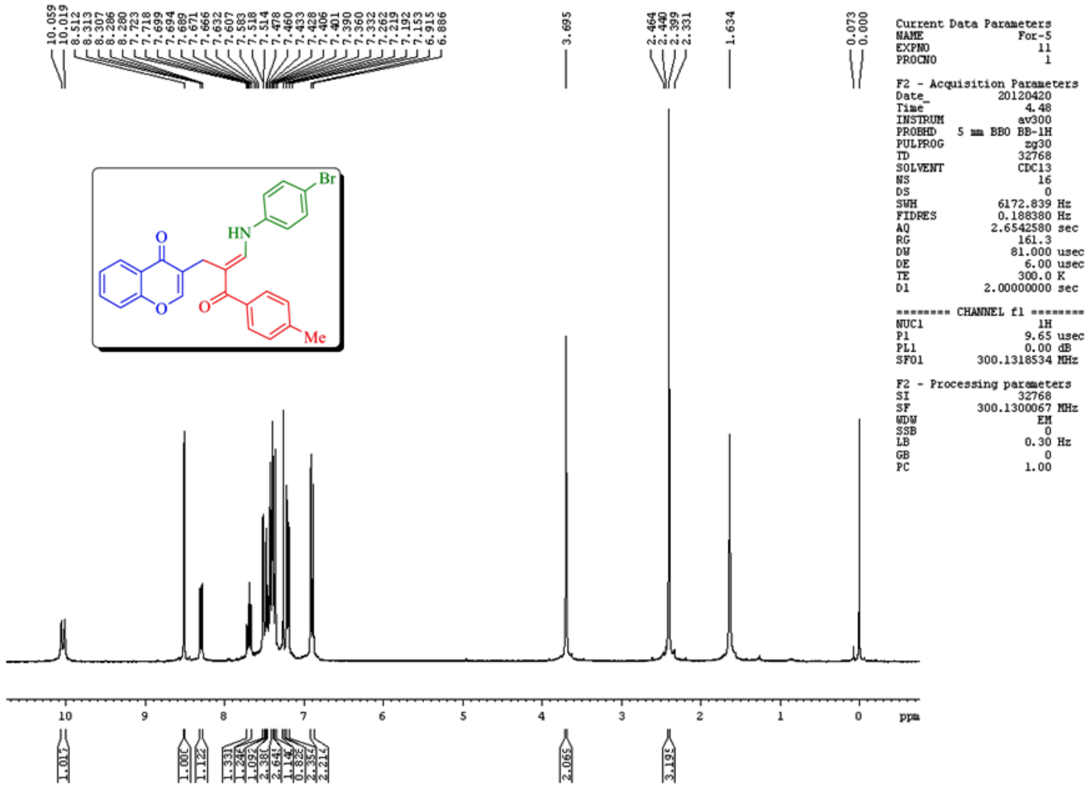

Figure 38. ^{1}**H NMR Spectrum of 5l** ($CDCl_3$)

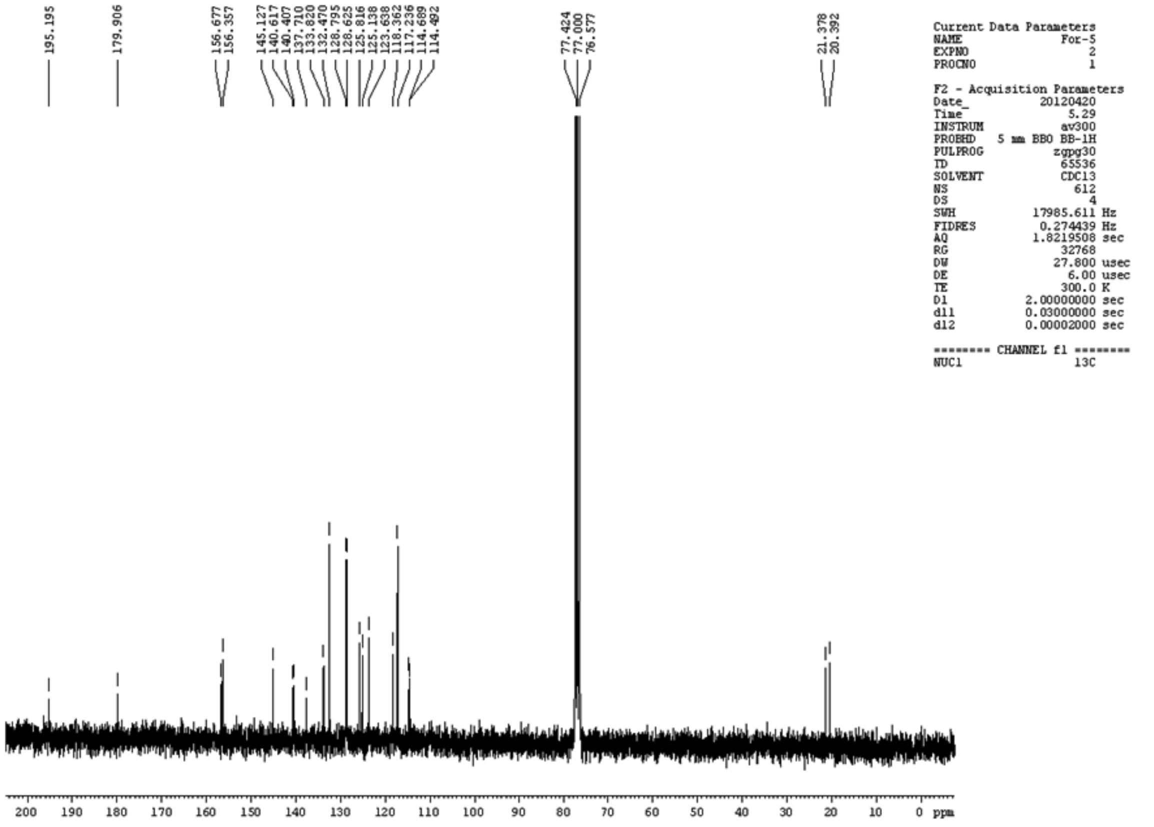

Figure 39. ^{13}**C NMR Spectrum of 5l (**CDCl$_3$)

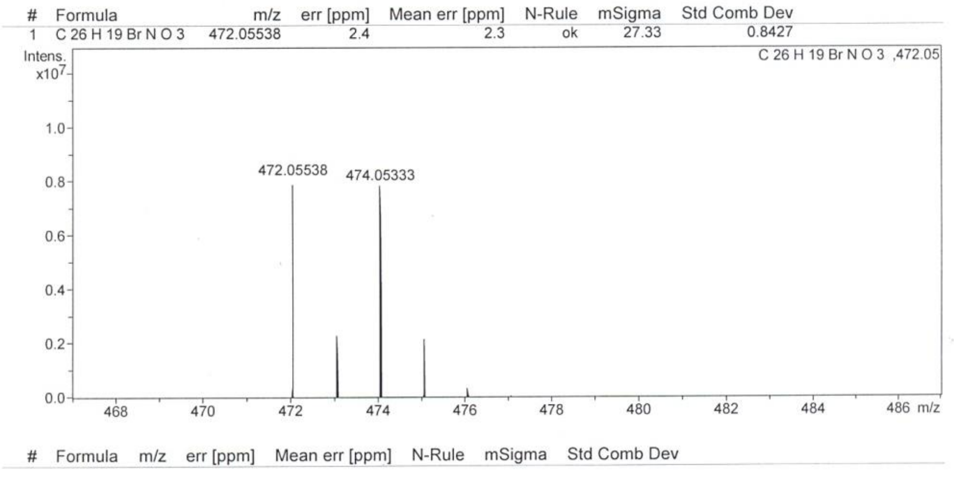

Figure 40. HRMS measurement for **5l**

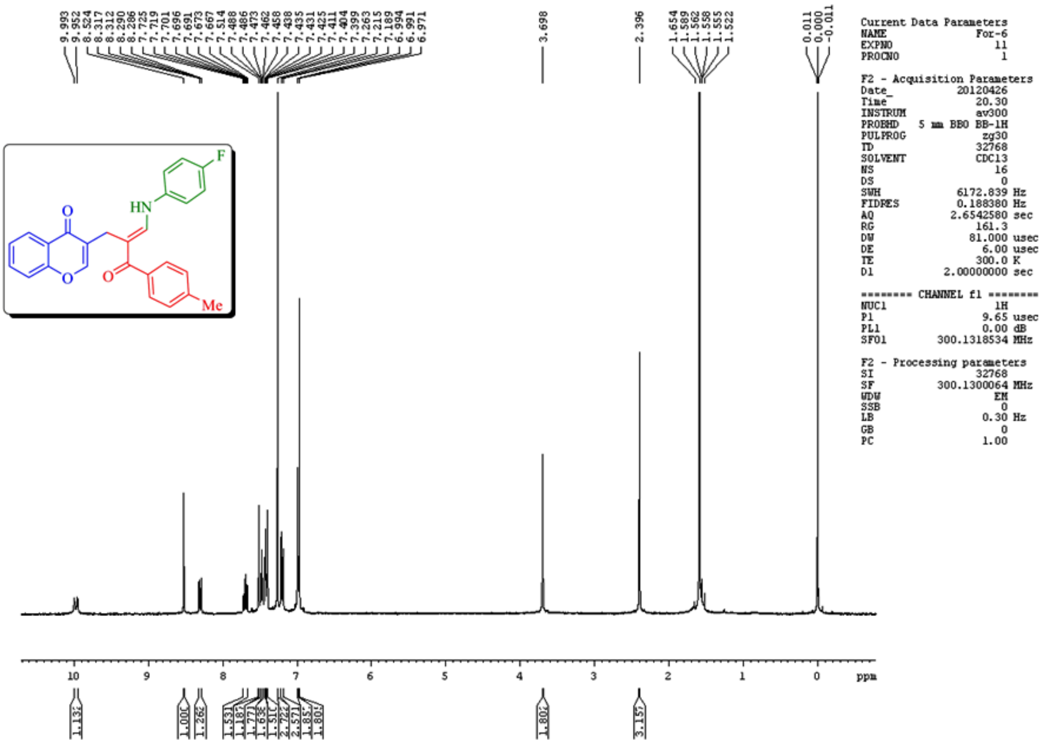

Figure 41. ^{1}H NMR Spectrum of 5m ($CDCl_3$)

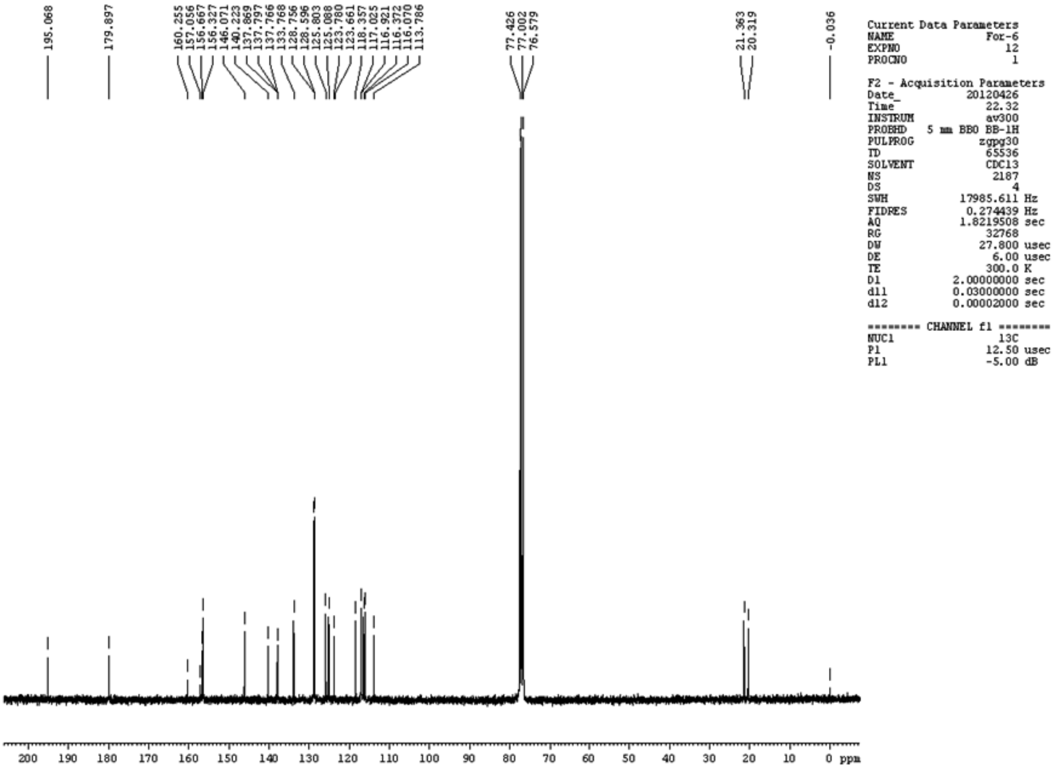

Figure 42. ^{13}C NMR Spectrum of 5m ($CDCl_3$)

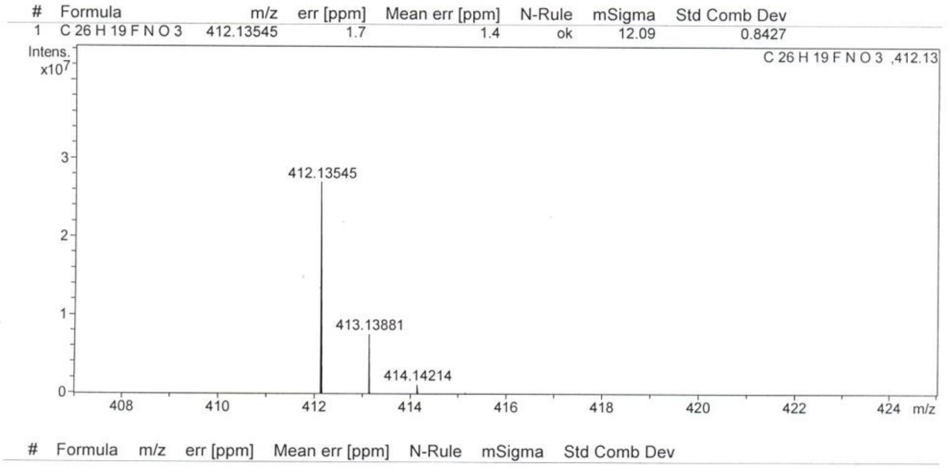

Figure 43. HRMS measurement for **5m**

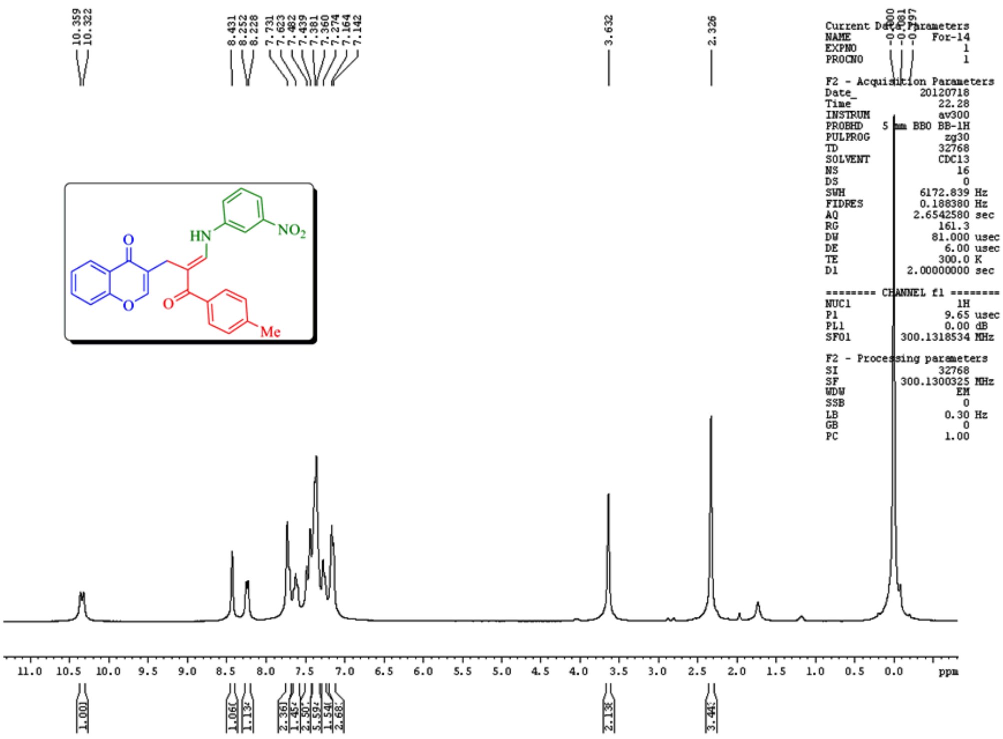

Figure 44. ^{1}H NMR Spectrum of 5n ($CDCl_3$)

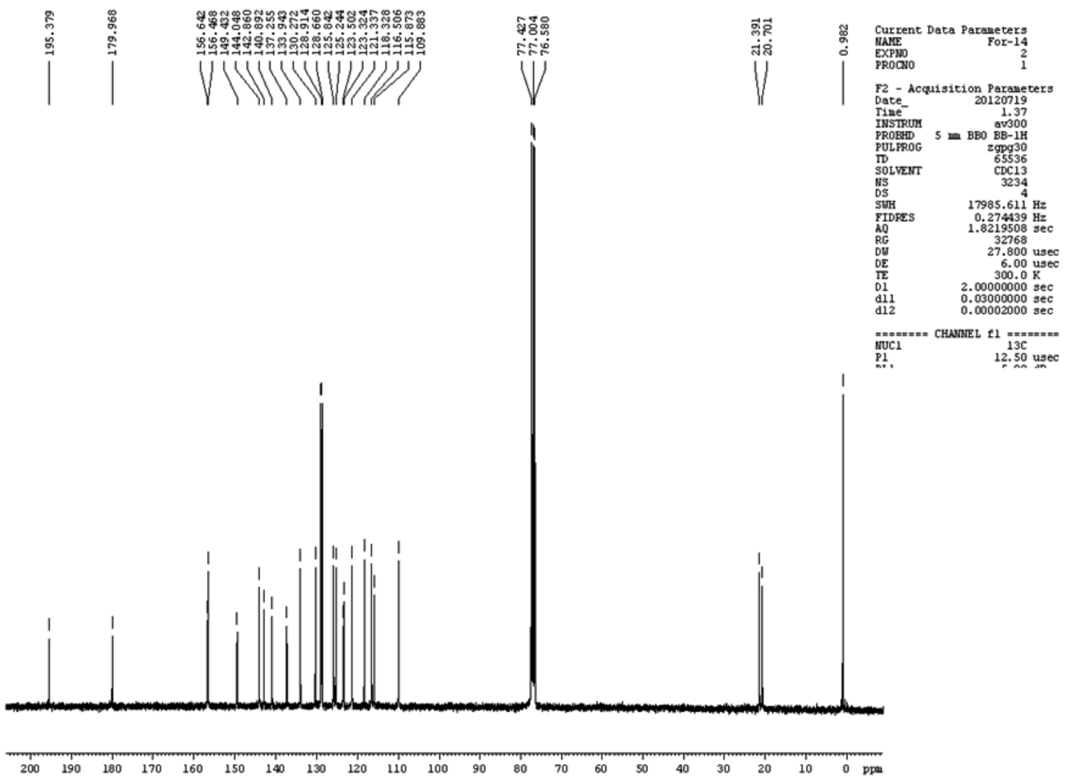

Figure 45. ^{13}**C NMR Spectrum of 5n (**$CDCl_3$)

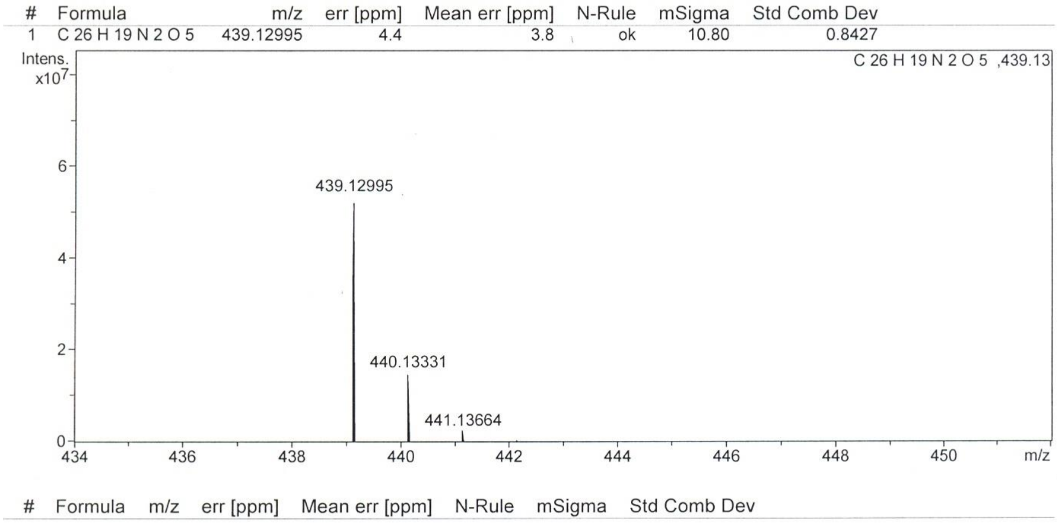

Figure 46.HRMS measurement for **5n**

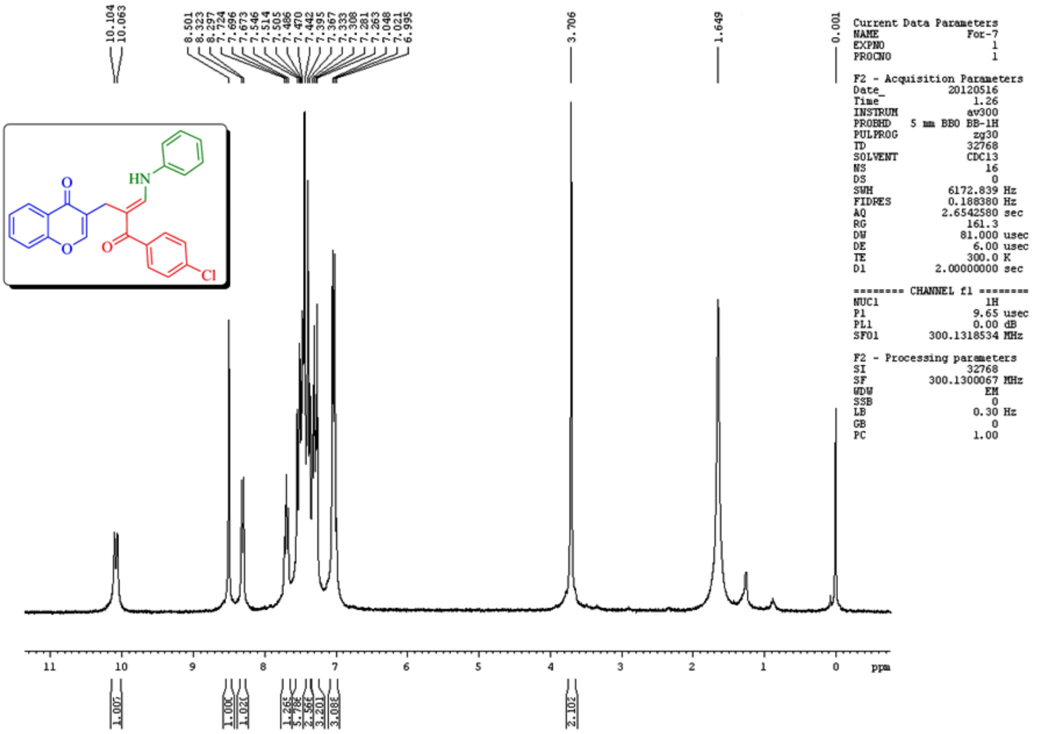

Figure 47. ^{1}**H NMR Spectrum of 5o** ($CDCl_3$)

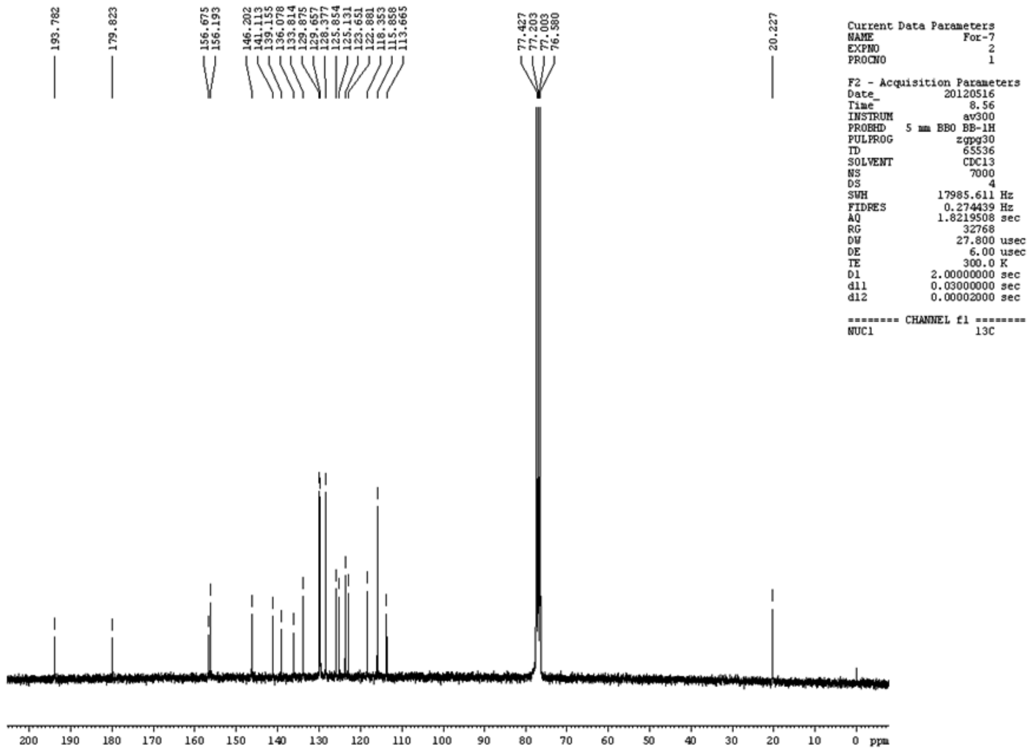

Figure 48. ^{13}**C NMR Spectrum of 5o** ($CDCl_3$)

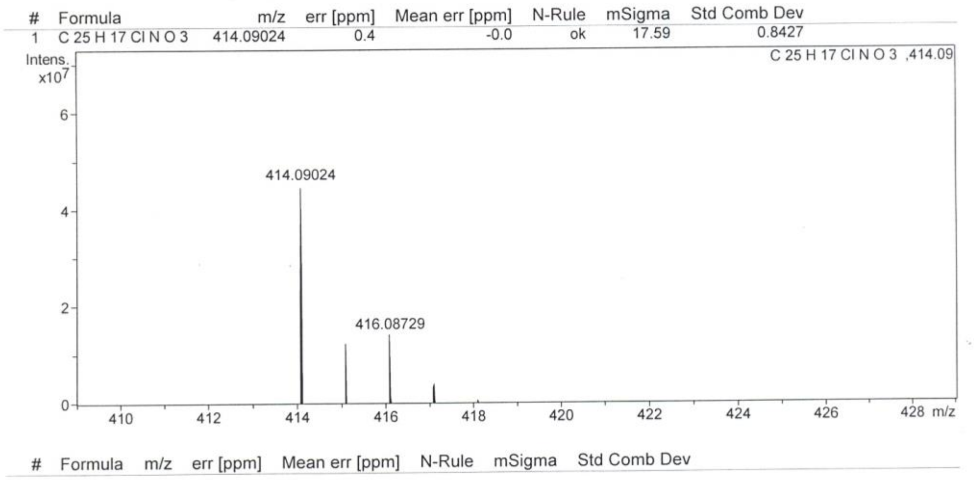

Figure 49. HRMS measurement for **5o**

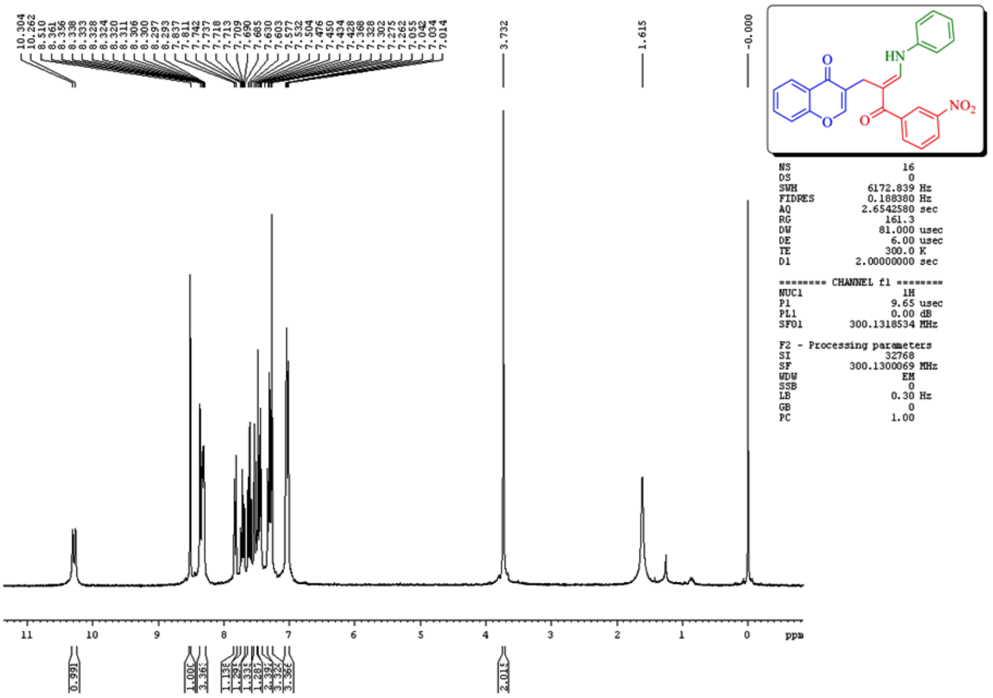

Figure 50. ^{1}H NMR Spectrum of 5p ($CDCl_3$)

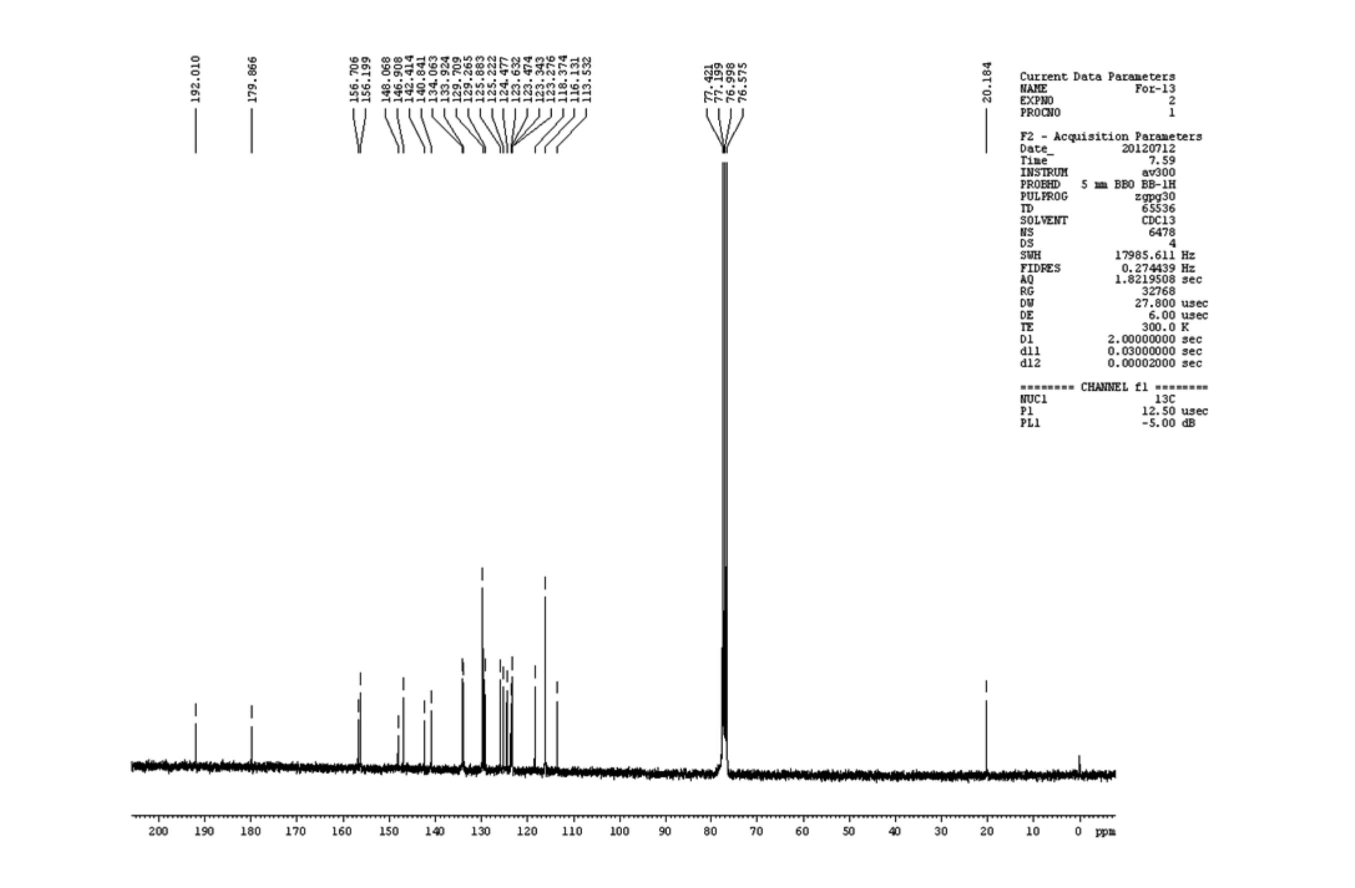

Figure 51. ^{13}**C NMR Spectrum of 5p (**$CDCl_3$)

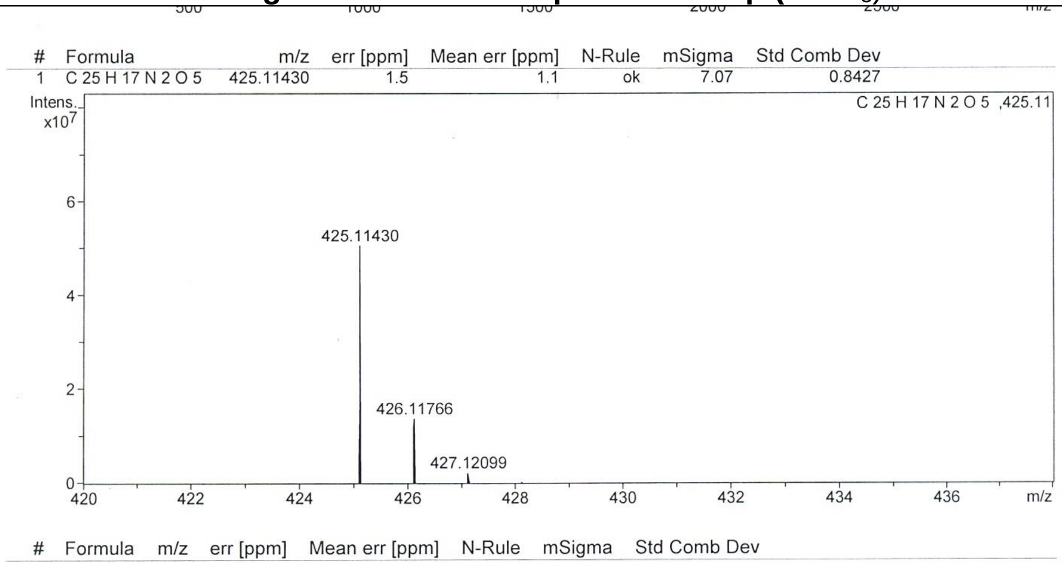

Figure 52. HRMS measurement for **5p**

Deuteration experiment

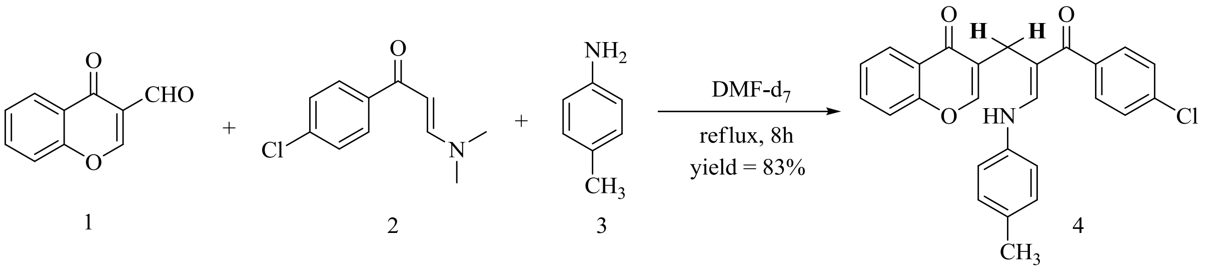

A mixture of 3-formylchromone (**1**, 0.1 mmol), enaminone **2** (0.1 mmol) and aniline **3** (0.1 mmol) in DMF-d_7 (1 mL) was heated to reflux for 6–7 h. The reaction progress was monitored by thin-layer chromatography. After completion of the reaction, the solvent was removed and the product was purified by column chromatography using a petroleum ether–ethyl acetate mixture (4:1 v/v) as eluent to afford compounds **4** in 83% of yield.

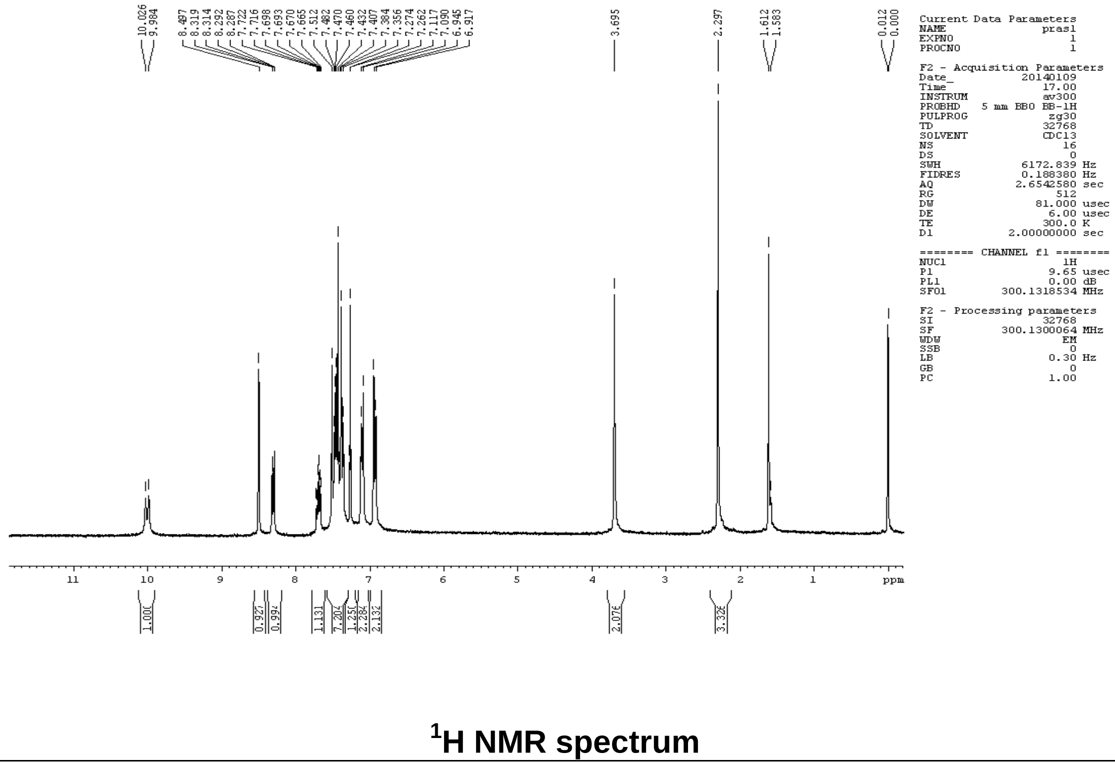

^{1}H NMR spectrum

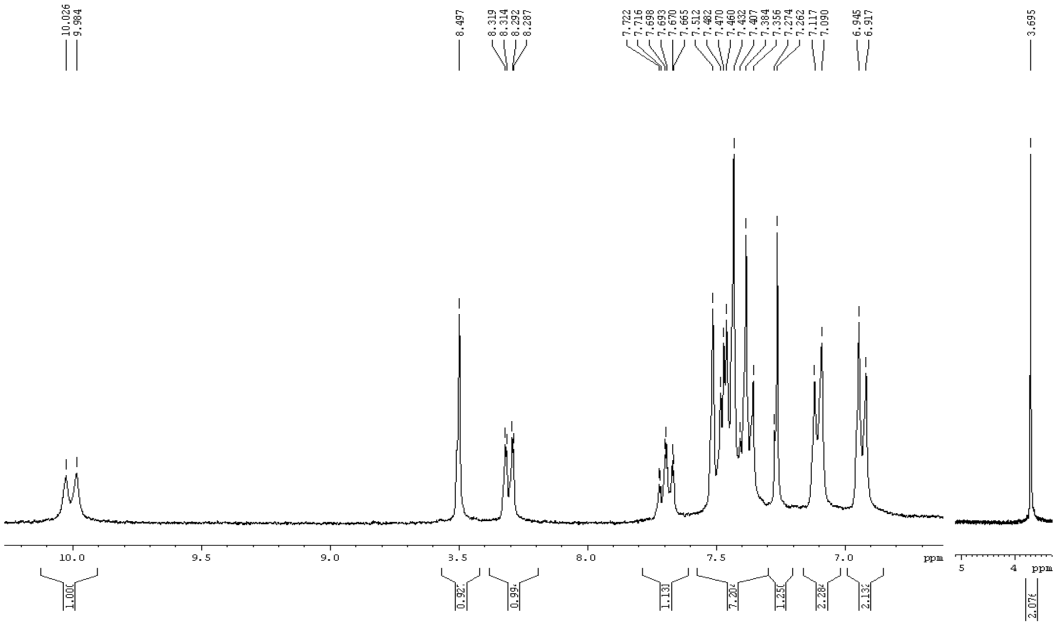

^{1}H NMR spectrum

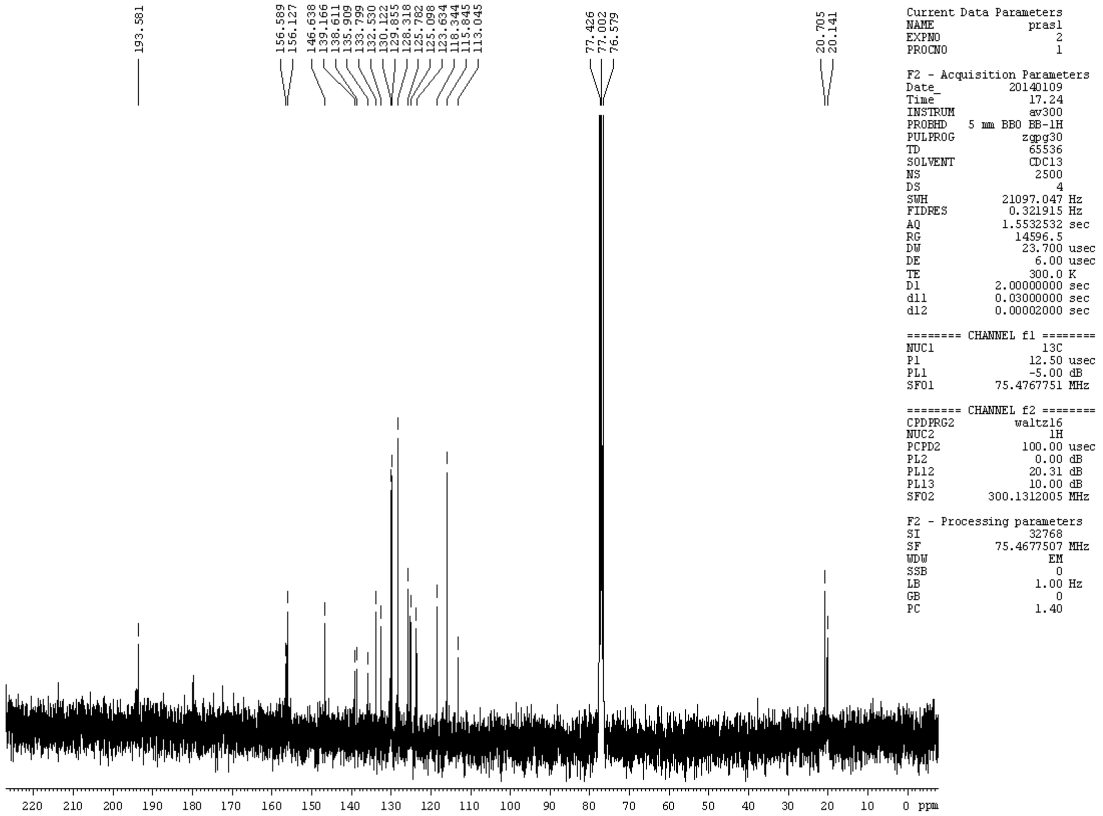

^{13}C NMR spectrum

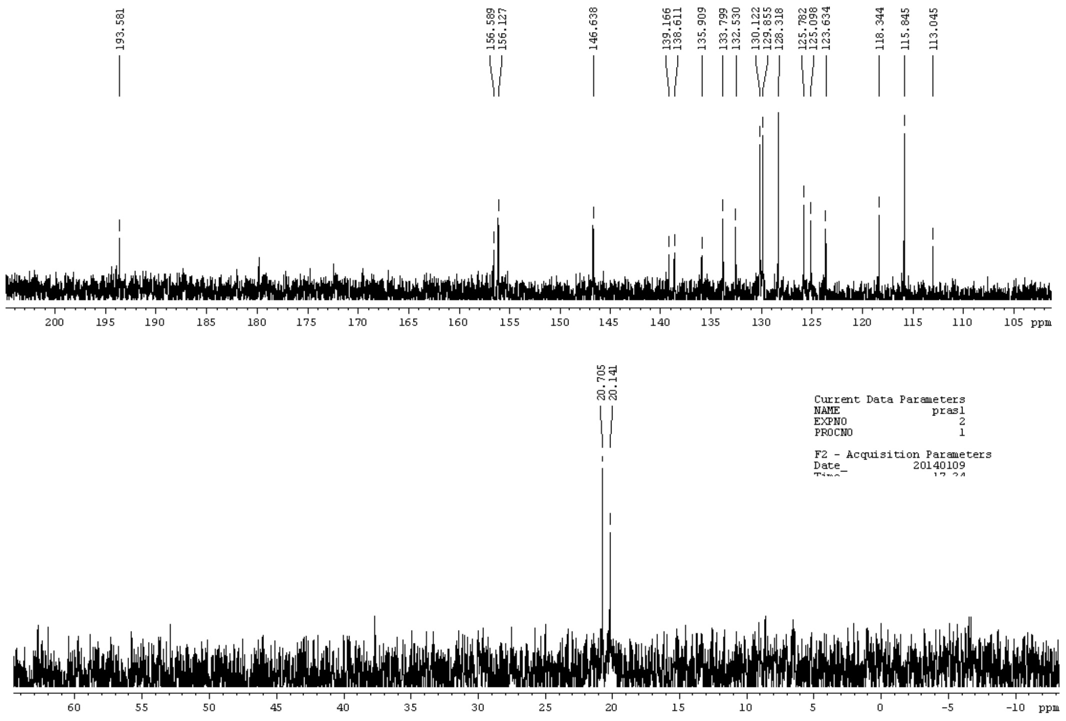

^{13}C NMR spectrum
